# Barriers to physical activity in patients with systemic lupus erythematosus in the UK

**DOI:** 10.1007/s10067-026-08324-3

**Published:** 2026-07-28

**Authors:** Aliaksandra Baranskaya, Lauren Franklin, Alex J. Wadley, John A. Reynolds

**Affiliations:** 1 Rheumatology Department, Sandwell and West Birmingham NHS Trust, Birmingham, UK; 2 Rheumatology Research Group, Department of Inflammation and Ageing, School of Infection, Inflammation and Immunology, College of Medicine and Health, University of Birmingham, Birmingham, UK; 3 School of Sport, Exercise, and Rehabilitation Sciences, College of Life and Environmental Sciences, University of Birmingham, Birmingham, UK

**Keywords:** Patient-reported outcomes, Physical activity, Quality of life, Systemic lupus erythematosus (SLE)

## Abstract

**Introduction:**

Physical activity (PA) may play an important role as a non-pharmacological addition to the management of SLE for disease control and reduction of cardiovascular risk factors. Those with SLE have been reported to engage in PA less than the general population.

**Objective:**

To describe PA patterns and patient-reported barriers to PA for people with SLE in the UK.

**Methods:**

An online survey was conducted of adults aged ≥ 18 years. Participants were recruited from posters in outpatient clinics, newsletters and Lupus UK social media platforms. Survey questions included demographic information, perception of disease activity, the International Physical Activity Questionnaire (IPAQ) and specific leisure time questions.

**Results:**

Two hundred sixty-eight patients participated, with a median (IQR) age of 51 (39–59) years and SLE disease duration of 10 (4–20) years were included. SLE-diagnosis was collected by self-report. In those with complete IPAQ data, 178/228 (79.1%) were in a moderate/high activity group. Participants in this group were more likely to be in employment and had lower levels of fatigue and pain. Fatigue was the most reported barrier to PA, irrespective of activity levels. Participants in the low PA group were more likely to have SLE-specific barriers such as higher disease activity and higher pain scores.

**Conclusions:**

Perceptions and preferences in relation to PA differ greatly between individuals with SLE. The most common self-reported barrier to PA was fatigue. Exploration of individual perceptions of PA should form part of consultations, to address perceived barriers.

**Table:** 

**Key Points** • *Some patients with self-reported SLE can meet WHO physical activity targets, especially those who remain in work.* • *Fatigue is the most frequently reported barrier to physical activity for patients, irrespective of their activity levels.* • *People are less likely to engage in PA if they believe it will negatively affect their SLE.*

**Supplementary Information:**

The online version contains supplementary material available at https://doi.org/10.1007/s10067-026-08324-3.

## Introduction

Systemic lupus erythematosus (SLE) is a chronic multisystem autoimmune disease which predominantly affects women. SLE is associated with significant physical morbidity and mortality, including cardiovascular disease (CVD) [1], and psychological morbidity including anxiety and depression [2].

Physical activity (PA) is an important intervention to improve outcomes of patients with inflammatory diseases. Self-reported measures of PA are commonly used in musculoskeletal research as they are relatively easy for patients to complete [3]. The International Physical Activity Questionnaire (IPAQ) is a widely used PA assessment tool. The IPAQ is advantageous as it allows categorisation of PA according to World Health Organisation (WHO) recommendations and provides descriptive data about the type of PA undertaken [4].

A study of just over 0.5 million people in the UK Biobank identified that those who engage in moderate and high levels of PA were less likely to develop SLE [5]. In patients with SLE, interventional studies have demonstrated that PA reduces CVD risk, fatigue and depression, and improves cardiorespiratory capacity and quality of life without increasing disease activity [6–14]. Reflecting this growing evidence base, the EULAR recommendations for non-pharmacological management of SLE recommend supporting patients to undertake PA in line with the WHO [15]. The WHO recommends at least 150 min of moderate-intensity PA or 75 min of high-intensity PA each week for the general population to improve cardiometabolic, musculoskeletal and cognitive health [16]. A recent European task force also supports the moderate-intensity PA target in patients with inactive or mild SLE [15], whilst patients with more active disease or flares should be assessed on an individual basis.Despite these recent guidelines, a systematic review identified that patients with SLE are less likely to meet the 150 min of weekly moderate PA targets than the general population and engage in more sedentary behaviours [6]. This behaviour is unlikely due to lack of knowledge of the benefits of PA, as patients with SLE report being aware of the benefits of PA to help manage their disease and well-being [19].

As reported levels of PA in the general population vary significantly between countries [20], it is highly likely that both levels of PA and barriers and facilitators to PA are context dependent. Several factors influence whether or not individuals engage in PA, extending from within-person factors, through inter-personal relationships and into the environment (the Socio-Ecological Model) [17]. Barriers are defined as by Garcia et al. (2022) as factors which “hinder, limit, or prevent people from engaging in a certain behaviour” whilst facilitators “favour, facilitate or help people to engage in a certain behaviour” [18].

AstEULAR recommends developing individualised exercise programmes to increase levels of PA in patients with SLE [21, 22], there is a need to understand current levels and types of PA undertaken and important facilitators and barriers experienced by patients with SLE in the UK.

## Methods

An online survey was conducted using Online Surveys (JISC) v.2 software licenced to the University of Birmingham. This online platform is GDPR complaint and certified to ISO 277001. Participants were eligible to take part in the survey if they were ≥ 18 years, living in the UK, and had a self-reported diagnosis of SLE. Participants were asked to confirm that the diagnosis of SLE was made by a medical professional.

Response to all questions, except for diagnosis confirmation, were non-compulsory. Participants self-reported all data within the survey. The English Index of Multiple Deprivation (IMD) 2019 [23], a measure of socioeconomic deprivation, was determined from postcodes for each participant residing in England. We were unable to calculate the IMD for respondents from Scotland, Wales or Northern Ireland.

SLE severity was defined by the Quick-Systemic Lupus Activity Questionnaire (Q-SLAQ) questionnaire [24]. The Q-SLAQ asks patients to rate common symptoms on a scale of 0–3 (not present, mild, moderate, severe) which are combined into a total score ranging from 0 to 34 where a higher number represents more active disease. The Q-SLAQ also asks patients to rate their lupus disease activity over the last 3 months on a scale from 0 (no activity) to 10 (most activity). PA was captured by participant responses to the long form version of International Physical Activity Questionnaire (IPAQ) [4]. The IPAQ questionnaire gathers data on PA across a seven-day period and splits this based on intensity (moderate vs vigorous) and activity domains (Work, Transportation, Leisure, Home and Garden). Participants self-reported the duration of time spent in each domain per day and frequency per week. Questions were included to investigate perceptions around PA and self-reported barriers. Specific leisure time PA questions were also included and were adapted from the nationally run Active Lives Survey by Sports England, an annual survey of children and adults in England focused on measuring PA engagement and attitudes [25].

A pilot survey was trialled by eleven patients with SLE, and their feedback was incorporated into the final survey version. A copy of the survey is contained in Supplementary Data File 1.

This study received a favourable ethical opinion from the South-East Scotland Research Ethics Committee (22/SS/0082) as a component of the EXamining the feasibility of exerCisE to manage symptoms of Lupus (EXCEL) study, REC [22/SS/0082]. All participants in the survey confirmed informed consent prior to undertaking the questionnaires and the study has been conducted according to the principles of the Declaration of Helsinki.

### Survey distribution

Patients who expressed interest during their outpatient clinic appointments at Sandwell and West Birmingham Hospital Trust, UK, were signposted to the survey via a QR code on a poster. Lupus UK social media platforms, the Lupus UK newsletter and the Patient Information Forum (PIF) newsletter, were also used to disseminate a direct link to the survey between June 2023 and October 2023. No financial incentives were offered to participants.

### Data processing

Duplicate entries with identical postcodes, ethnicity, age, sex and body mass index (BMI) were deemed to be from same individual and the records with the most complete data were retained.

Answers to the IPAQ questionnaire from participants aged 18–69 were processed as per IPAQ Guidelines for Data Processing and Analysis (2005) which included exclusion of participants over 69 years old, removal of outliers with > 960 min of daily activity, recoding responses of activity bouts lasting < 10 min as 0 min and limiting time to a maximum 180 min per activity per day [26]. Participants were included in IPAQ analysis if ≥ 2/3 responses were recorded in each IPAQ domain and missing responses were considered as “no activity”. PA levels derived from IPAQ responses were reported as both continuous and categorical variables. Continuous data comprised metabolic equivalent of task (METs) minutes per week, a commonly used measure of PA intensity against resting metabolic rate [27]. Cycling and vigorous garden activity were treated as moderate intensity in METs calculations and in categorisation.

Patients were grouped into low PA (< 150 min of moderate or < 75 min of vigorous PA per week) or moderate/high based (≥ 150 min of moderate or ≥ 75 min of vigorous PA per week) as per the WHO PA recommendations (which is equivalent to ~ 500–1000 METs per week [28]).

Additional questions on the different types of leisure time PA were processed as per Active Lives Survey methodology [29]. Activity was counted if it was equivalent to 30 min activity at least twice in 28 days, with each session lasting at least 10 min and of at least moderate intensity.

### Statistical analysis

Data are presented as either *n* (%), or median (IQR). Non-parametric tests (Chi-square and Mann–Whitney *U* test) were used for categorical and continuous variables respectively. If the expected frequency within cells was low, Fisher’s exact test was used as preference. *P*-values below 0.05 were deemed statistically significant. To quantify the association between variables, univariate and multivariable logistic regression models were used. As the diagnosis of SLE was self-reported and as most patients would be expected to be taking hydroxychloroquine, a sensitivity analysis was conducted in which only participants who reported taking antimalarials were included. Statistical analysis was undertaken using Stata/SE v18.0.

## Results

Data were collected between 5th May and 19th October 2023. After removal of duplicate entries, there were 268 responses available for analysis. The characteristics of respondents are shown in Table 1. Most participants were of White ethnicity (229/268, 85.4%) and female (258/267, 96.3%). The median (IQR) age was 51 (39–59) years with a disease duration of 10 (4–20) years. Participants had a median BMI of 27.1 (23.5–32.1) kg/m^2^. Participants from all four UK nations were recruited with the vast majority residing in England (216/256, 84.4%). Most participants had attained at least a degree or equivalent level of education (169/259, 65.3%) and just over half were in current employment (145/264, 54.9%). In England, there was representation from all socioeconomic quintiles but 51.4% (110/214) resided in IMD quintiles 4 and 5 (the least deprived areas). The most frequent medications reported were antimalarials (216/268, 80.6%) and oral prednisolone (87/268, 32.5%). Just under 10% (21/268, 7.8%) reported receiving intravenous therapy (belimumab or IV steroids) in last 6 weeks, or rituximab/cyclophosphamide in last 12 months. Self-reported disease activity over the preceding 3 months was 5 (2–7). The median Q-SLAQ score from 243 individuals with a valid score was 14 (10–19). Reported medication use and co-morbidities are shown in Supplementary Tables 1 and 2.

**Table 1:** Characteristics of survey respondents

	Median (IQR) or *n* (%)
Sex: Female (*n* = 267)	258 (96.3%)
Age (years)	51 (39–59)
Ethnicity	
White	229 (85.4%)
Asian (including Chinese)	11 (4.1%)
Black	9 (3.4%)
Mixed	12 (4.5%)
Other Ethnic Group	7 (2.6%)
UK Country (from postcode) (*n* = 256)	
England	216 (84.4%)
Scotland	19 (7.4%)
Wales	16 (6.3%)
Northern Ireland	5 (2.0%)
Socioeconomic Deprivation based on English IMD 2019 (*n* = 214)	
IMD quintile 1 (most deprived)	20 (9.3%)
IMD quintile 2	43 (20.1%)
IMD quintile 3	41 (19.2%)
IMD quintile 4	63 (29.4%)
IMD quintile 5 (least deprived)	47 (22.0%)
Lupus disease features	
Disease duration (years) (*n* = 263)	10 (4–20)
Total Q-SLAQ score (*n* = 243)	14 (10–19)
Self-reported lupus disease activity (0–10) (*n* = 263)	5 (2–7)
Lifestyle	
BMI (kg/m^2^): (*n* = 261)	27.1 (23.5–32.1)
Smoking status (*n* = 265)	
Current tobacco smoker	12 (4.5%)
Current e-cigarette/vape smoker Smokes both tobaccos and e-cigarette/vape	12 (4.5%) 2 (0.8%)
Alcohol consumption (Yes): (*n* = 267)	130 (48.7%)
Units per week (in those answering yes to above) (n = 125)	5 (2.3–8.0)
Employment status (*n* = 264)	
Working (full or part time)	145 (54.9%)
Unemployed	4 (1.5%)
Retired	46 (17.4%)
Sick/disabled	46 (17.4%)
Carer/homemaker	11 (4.2%)
Student	7 (2.7%)
Other	5 (1.9%)
Highest educational attainment (*n* = 259)	
No secondary education qualification	11 (4.2%)
Secondary education	28 (10.8%)
Higher secondary	51 (19.7%)
Degree or equivalent or above	169 (65.3%)
Quality of life	
Pain in the past week (0–10) (*n* = 264)	5 (2–7)
Unusual fatigue in the past week (0–10) (*n* = 266)	7 (4–8)
Problems sleeping in the past week (0–10) (*n* = 266)	6 (3–8)
Mood in the past week (0–10) (*n* = 264)	5 (3–7)

### Physical activity levels in patients with SLE

There were 228/268 (85.1%) valid IPAQ responses for analysis. Of the 40 patients excluded, 22 were due to age > 69 years, 8 reported > 960 min of PA and the remainder were excluded due to incomplete data. The characteristics of all excluded patients, and those excluded due to the age criterion are shown in Supplementary Table 3.

The results of the PA levels from the IPAQ are shown in Table 2. The median weekly METS for all activity was 2423 (861–4885), with 676.5 (198–1980) for walking, 825 (255–2165) for moderate PA and 0 (0–480) for vigorous PA. Across different IPAQ Domains, highest engagement (proportion of respondents reporting > 0 min) was seen in Home and Garden based activities (187/228, 82.0%). The lowest engagement was seen in Work Based activities (70/228, 30.7%), but amongst those in work, this domain had the highest median METS and total weekly minutes (Table 2). In terms of sport or leisure time PA, walking was most frequent with 98/260 (37.7%) reporting at least one episode compared to dedicated sport activities (28/261, 10.7%), cycling (18/264, 6.8%), fitness-based exercise (12/263, 4.6%) and dance (11/262, 4.2%) (details of preferred types of exercise are shown in Supplementary Tables 4 and 5).

**Table 2:** Characteristics of PA reported in the IPAQ from 228 participants

Characteristic	Median (IQR) or *n* (%)
**Weekly METS**	
Total Walking	676.5 (198–1980)
Total Moderate activity	825 (255–2165)
Total Vigorous activity	0 (0–480)
Total All	2422.5 (861–4885)
**IPAQ activity domains**	
***Work***	
Number with > 0 weekly mins	70 (30.7%)
Median METS*	1634 (480–3798)
Median total weekly minutes*	372.5 (120–900)
***Travel (walking and cycling only)***	
Number with > 0 weekly mins	164 (71.9%)
Median METS*	396 (198–1026)
Median total weekly minutes*	120 (60–300)
***Home and garden***	
Number with > 0 weekly mins	187 (82.0%)
Median METS*	720 (360–2160)
Median total weekly minutes*	240 (90–570)
***Leisure***	
Number with > 0 weekly mins	164 (71.9%)
Median METS*	792 (325–1782)
Median total weekly minutes*	210 (90–420)
**IPAQ PA categories**	
Low	50 (21.9%)
Moderate	74 (32.5%)
High	104 (45.6%)

^*^if participant reports > 0 min weekly

79.1% (178/228) of respondents were in the moderate/high PA group. By country, 77.3% (143/185) respondents in England, 84.6% (11/13) in Wales, 93.8% (15/16) in Scotland and 80.0% (4/5) in Northern Ireland were in the moderate/high PA group. As the survey relies on self-reported classification of SLE diagnosis, a sensitivity analysis was undertaken using only those individuals who reported taking hydroxychloroquine with a valid IPAQ (*N* = 189). In this group, 19% (36/189) were classified into the low PA group, 34.4% (65/189) in moderate and 46.6% (88/189) in high. The median total weekly METs were 2471 (927–4857).

When compared to individuals in the low PA group, those in the moderate/high PA group had higher percentage engagement in all activity domains (see Table 3). Only 21/50 (42%) of those in low PA category engaged in leisure-based PA with 40 median weekly minutes (20–60) compared to 143/178 (80.3%) of those in the mod/high category (median 240 [120–480] weekly minutes).

**Table 3:** Reported engagement in each PA domain and the median minutes for each activity domain per week split by PA category

IPAQ Domain	Low PA N = 50 (%)	Moderate/High PA N = 178 (%)	*p*-value (Chi-square or Mann–Whitney *U* test)
**Work**	2 (4%)	68 (38.2%)	< 0.001
	25 (20–30)*	387.5 (150–900)*	0.010
**Leisure**	21 (42%)	143 (80.3%)	< 0.001
	40 (20–60)*	240 (120–480)*	< 0.001
**Travel**	25 (50%)	139 (78.1%)	< 0.001
	30 (15–60)*	180 (60–375)*	< 0.001
**Garden and Home**	27 (54%)	160 (89.9%)	< 0.001
	50 (30–120)*	285 (127.5–660)*	< 0.001

^*^in those reporting > 0 min per week

When asked to rate satisfaction with current PA levels (on a scale of 0–10, where 0 represents very unsatisfied), participants in the low PA category reported significantly lower levels of satisfaction than others (median score 0 [0–1] vs 3 [1–6], *p* < 0.001). The preferred setting for PA also differed significantly between participants in low PA group compared to those in the moderate/high PA group. Participants with low PA preferred home-based PA (21/50, 42.0%) followed by outside settings (13/50, 26.0%), and indoor facilities such as gym (8/50, 16.0%) whilst those in the moderate/high PA group rated outside settings as most preferable (71/178, 39.9%), followed by home (39/178, 21.9%), and indoor facilities (36/178, 20.2%) (*p* = 0.037).

### Factors associated with PA

There were more males in the low PA group (3/178 [1.7%] vs. 4/50 [8.0%], *p* = 0.007) but no significant differences in age, ethnicity, education or IMD between the groups (see Table 4). Patients fulfilling WHO targets had a significantly lower BMI (26 vs. 32 kg/m^2^, *p* < 0.0004). Patients in the moderate/high PA group were more often in employment (64.6% vs. 38.0%, *p* = 0.028) compared to in the low PA group. In terms of SLE features, participants in the low PA category reported significantly higher disease activity, pain and fatigue scores (see Table 4).

**Table 4:** Characteristics of those in Low PA category versus moderate/high category

	MOD/HIGH PA *n* = 178	LOW PA *n* = 50	***p***-value*
	Median (IQR) or *n* (%)		
Sex (female)	175 (98.3%)	46 (92.0%)	0.007
Age (years)	48 (38–57)	51(36–59)	0.724
Ethnicity (White)	155 (87.1%)	41 (82%)	0.361
BMI (kg/m^2^)	26 (23.3–30.2)	32 (25.4–34.9)	< 0.001
IMD quintile	*n* = 141	*n* = 42	0.935
1 (most deprived)	16 (11.4%)	4 (9.5%)	
2	29 (20.6%)	7 (16.7%)	
3	28 (19.9%)	8 (19.1%)	
4	35 (24.8%)	13 (31%)	
5 (least deprived)	33 (23.4%)	10 (23.8%)	
Educational attainment	*n* = 173	*n* = 49	0.700
No secondary education	6 (3.5%)	1 (2.0%)	
Secondary education	16 (9.3%)	6 (12.2%)	
Higher secondary	33 (19.1%)	12 (24.5%)	
Degree or equivalent or above	118 (68.2%)	30 (61.2%)	
Part- or full-time employment	113/175 (64.6%)	19 (38.0%)	0.001
Q-SLAQ (0–34)	14 (9–18)	15 (13–20)	0.025
Disease duration (years)	10 (3–20)	10 (5–18)	0.744
Pain over past week**	4 (2–7)	6 (5–8)	0.002
Fatigue over past week**	6 (3–8)	8 (6–9)	0.004
Reported use of activity tracking device	109/176 (61.9%)	24 (48%)	0.077
Use of daily step target goal	91/108 (84.3%)	15/24 (62.5%)	0.015

^*^P-values have been generated from Chi^2^ and Mann–Whitney *U* test, for categorical and continuous variables respectively

^**^on a scale of 0–10 when 10 is worst

In logistic regression models (adjusted for sex and age), higher BMI (OR 0.94 [0.90,0.98]), unemployment (OR 0.34 [0.17,0.67]), Q-SLAQ score (OR 0.94 [0.89, 0.99], fatigue (OR 0.83 [0.73, 0.94]) and pain scores (OR 0.81 [0.72, 0.92] were inversely associated with being in the moderate/high PA group. In a model also adjusting for BMI, disease activity (QSLAQ) and social deprivation, the associations with unemployment and remained statistically significant (Table 5).

**Table 5:** Univariate and multivariable regression models of likelihood of being in the moderate/high category

		Univariate		Adjusted for age and sex		Full model*	
	n	OR (95% CI)	*p*-value	OR (95% CI)	*p*-value	OR (95% CI)	*p*-value
Sex (male)	227	0.13 (0.02, 0.74)	0.021	-	-		
Age	228	1.00 (0.97, 1.02)	0.724	-	-		
BMI (kg/m^2^)	222	0.94 (0.91, 0.98)	0.002	0.94 (0.90, 0.98)	0.002		
IMD quintile	183						
1 (most deprived)		*ref*	-	*ref*	-		
2		1.04 (0.26, 4.08)	0.960	1.00 (0.25, 3.96)	0.998		
3		0.88 (0.26, 3.37)	0.846	0.92 (0.24, 3.55)	0.900		
4		0.67 (0.19, 2.39)	0.540	0.71 (0.20, 2.56)	0.605		
5 (least deprived)		0.83 (0.22, 3.04)	0.773	0.86 (0.23, 3.20)	0.824		
Disease duration (years)	225	1.01 (0.98, 1.04)	0.531	1.01 (0.98, 1.04)	0.615	1.01 (0.98, 1.06)	0.394
QSLAQ	213	0.94 (0.90, 0.99)	0.020	0.94 (0.89, 0.99)	0.015		
Unemployed	225	0.34 (0.18, 0.64)	0.001	0.34 (0.17, 0.67)	0.002	0.39 (0.16, 0.87)	0.023
Fatigue	227	0.84 (0.75, 0.95)	0.005	0.83 (0.73, 0.94)	0.003	0.86 (0.71, 1.03)	0.092
Pain	225	0.83 (0.74, 0.94)	0.002	0.81 (0.72,0.92)	< 0.001	0.82 (0.69, 0.98)	0.027
Satisfaction with PA	224	1.44 (1.22, 1.71)	< 0.001	1.48 (1.23, 1.77)	< 0.001	1.74 (1.32, 2.29)	< 0.001
Step goal	226	1.76 (0.94, 3.32)	0.079	1.67 (0.87, 3.20)	0.120	3.00 (0.73, 12.9)	0.134

*adjusted for age, sex, BMI, QSLAQ score and IMD

Patients in the moderate/high PA group were more likely to have positive perceptions around the effect of PA on SLE (OR 1.36 [1.18,1.57]) and higher satisfaction with current PA levels (OR 1.48 [1.23,1.77]). Respondents in the low PA category were more likely to think that PA would worsen SLE symptoms (median score 3 [1–5] vs 5 [4–7], *p* < 0.001) (on a 0–10 scale where 0 represents significant worsening of symptoms) (Table 5).

### Potential facilitators of PA in patients with SLE

Whilst reported use of activity tracking devices (such as smart watches) did not differ significantly between the groups, a higher proportion of those in the moderate/high PA group used a daily step target goal (*p* = 0.015) (see Table 4).

Across all respondents, 25.9% (69/266) reported previously receiving personal training (PT). There was no difference in previous PT use between the low and moderate/high PA group (24% vs. 31%, *p* = 0.333), however participants reported a positive experience of PT (median score 6 [4–9], on a 0–10 scale where 0 is very unsatisfactory and is 10 very satisfactory) with higher scores reported by patients in the moderate/high PA group (7 [5–9) vs 4 [2–7], *p* = 0.0165).

### Self-reported barriers to PA

Fatigue was reported as barrier to PA by 236/268 (88.1%) respondents (see Table 6). When participants were restricted to reporting a single barrier, fatigue was also the most frequently chosen across all activity groups (see Supplementary Table 6). Pain and disease flares were also frequently reported by majority of participants.

**Table 6:** Self-reported barriers to PA according to the IPAQ PA category in response to the question *“What prevents you from carrying out more leisure time exercise/physical activity?”*

	Whole cohort *n* = 268	IPAQ PA category		*p*-value (Chi-square test of low vs. mod/high)
		Low (*n* = 50)	Moderate/High(*n* = 178)	
Caregiving	23 (8.6%)	5 (10.0%)	18 (10.1%)	0.981
Concern for injury	41 (15.3%)	9 (18.0%)	28 (15.7%)	0.701
Concern for lupus worsening	67 (25.0%)	21 (42.0%)	41 (23.0%)	0.008*
Dislike of exercise	26 (9.7%)	11 (22.0%)	11 (6.2%)	0.001*
Fatigue	236 (88.1%)	49 (98.0%)	156 (87.6%)	0.032
Financial strains	34 (12.7%)	11 (22.0%)	20 (11.2%)	0.050
Lupus flare	155 (57.8%)	36 (72.0%)	105 (59.0%)	0.094
Other illness	90 (33.6%)	25 (50.0%)	47 (26.4%)	0.002*
Facilities	20 (7.5%)	6 (12.0%)	11 (6.2%)	0.166
Knowledge	24 (9.0%)	8 (16.0%)	15 (8.4%)	0.116
Motivation	90 (33.6%)	20 (40.0%)	50 (28.1%)	0.410
Time	81 (30.2%)	6 (12%)	70 (39.3%)	< 0.001*
Pain	166 (61.9%)	37 (74.0%)	109 (61.2%)	0.097
Self-consciousness	51 (19.0%)	15 (30.0%)	30 (16.9%)	0.039
Weather	60 (22.4%)	11 (22.0%)	39 (21.9%)	0.989
Other	17 (6.3%)	4 (8.0%)	10 (5.6%)	0.535

^*^Statistically significant after Benjamini–Hochberg correction

Some barriers differed between low and medium/high PA groups. In an exploratory analysis, those in the low PA group more frequently reported fatigue, other illnesses, and self-consciousness than other participants. Negative perceptions of PA were more likely to be reported by those in low PA groups including concern that SLE may be worsened by PA and dislike of exercise. After correction for multiple comparisons, patients in the low PA group were more likely to report concerns about lupus worsening, or other illnesses and state that they disliked exercise; they were less likely to report time as a limiting factor (see Table 6).

The reporting of some barriers to PA varied by participant age, BMI and disease duration. Younger participants reported concern for injury, SLE flares and time as barriers. Participants who were concerned about injury had a shorter disease duration (*p* = 0.0005) and those concerned about lupus flares were younger (*p* = 0.0007) with shorter disease duration (*p* = 0.0012). Participants reporting time as a barrier were also younger (*p* = 0.0001). Participants reporting pain (*p* = 0.003), fatigue (*p* = 0.0014) and self-consciousness (*p* < 0.0001) all had a higher BMI (see supplementary Table 7).

## Discussion

This study reports the PA habits of patients with SLE in the UK and has identified important differences between patients who undertake lower or moderate/high PA in terms of types of PA and barriers to undertaking PA.

Our finding that 79.1% of individuals with self-reported SLE engaged in sufficient PA to meet weekly WHO targets is greater than the 64% reported in the general population in the Health Survey for England 2021 [30]. In the UK Biobank, 64% of participants with inflammatory rheumatic diseases met the target compared to 74% of healthy participants [31]. Our study population has key differences from the general population as they were predominantly female, educated and living in areas of low socio-economic deprivation which makes it difficult to compare between our participants and the general population directly. In studies with a direct comparator, patients with SLE report lower levels of PA compared to healthy controls [32].

If we consider attainment of PA activity recommendations, there is significant heterogeneity in the rates of PA target fulfilment in patients with SLE. A recent systematic review by Blaess et al. (2023) described PA target fulfilment rates of 28% to 89.5% using self-reported measures and 11% to 29.8% using objective measures [6]. These data indicate that patients may over-estimate their PA levels on self-reported questionnaires. Similarly, in a direct comparison, the IPAQ score only modestly correlated with PA assessed by accelerometer in patients with SLE [33]. Compared to a recent international survey using the short-form IPAQ, more patients in our study had moderate or high levels of PA based on IPAQ (2423 METs/week vs. 936 METs/week) [34]. This may be due to differences between countries, as a cross-sectional survey in Germany reported a mean of 3028 METs/week in 146 patients with SLE [35]. There is, however, also evidence that the short-form IPAQ poorly correlates with the long-form used in our study and may under-estimate PA [36].

We found that patients meeting moderate activity targets had a lower BMI, higher satisfaction with current level of leisure time PA, and had positive perceptions around the effects of PA, specifically in relation to disease activity. Conversely, higher disease activity and fatigue scores were associated with low levels of PA. This is comparable to work by others which show that patients with more active disease and related fatigue report lower self-reported PA [37, 38]. It is important to recognise that due to the cross-sectional design of the study is it not possible to infer causality, for example fatigue may be both a reason for low PA and a consequence of low PA. Interestingly, in contrast to the Health Survey for England 2021, patients in this study reporting lower PA were more likely to be men.

Patients who met moderate or high activity targets were more often employed and, in those patients, work and travel comprised much of their PA time. These patients were also more likely to undertake PA in their leisure time although work-based PA may already be sufficient to meet their targets. Whilst this is encouraging, a recent umbrella review identified that although work-based PA may be beneficial for some health outcomes (e.g. cardiovascular disease), it is also associated with poor health outcomes including osteoarthritis, depression and anxiety, and poor sleep quality [39]. Therefore, it may be important to encourage leisure time PA in patients with SLE even if they have significant occupational PA.

We identified key barriers to PA experiences by patients with SLE. Across all respondents, fatigue was the most frequent barrier (88%), irrespective of participants’ self-reported level of PA. Fatigue affects around 80% people with SLE [40] and is therefore an important barrier. Patients with low PA reported more disease-specific barriers such as higher disease activity and higher pain scores. Participants who have been more recently diagnosed were more likely to be worried about PA making SLE disease worse or causing SLE flare, as has been reported by others [19]; highlighting an important opportunity to better educate patients about the benefits of PA early in their disease course. Our study supports others which have identified that adverse weather, health concerns, dislike of exercise, financial constraints and work demands (lack of time) are all barriers to PA in SLE [41, 42]. Interestingly, time was less likely to be reported as a barrier in patients with low levels of PA. This may be because patients who don’t really participate in PA don’t often consider how much time PA might take.

A key limitation of our study is that SLE diagnosis was self-reported and so participants may not have SLE (but have other related conditions such as cutaneous lupus or other systemic autoimmune diseases). We aimed to explore this by conducting a sensitivity analysis using only individuals reporting hydroxychloroquine. Whilst this is not intended to be a substitution for confirmed physician diagnosis, the results showed comparable results to the whole study population.

In addition, the participants may not fully reflect all patients with SLE in the UK or worldwide. Although our study population is comparable to other survey populations, in terms of ethnic distribution and levels of employment [40, 43], it is well-recognised that patients from ethnic minority backgrounds are often under-represented in research studies. Our approach to recruitment and the use of Lupus UK-associated social media may not have reached under-represented communities. It is important that future studies attempt to recruit these populations to capture key differences in behaviour and health beliefs. Our cohort also had a greater proportion of individuals of higher socioeconomic status and educational attainment, which suggests that there was selection bias in the population, which may limit the generalisability of our results and make robust comparisons with general population data difficult; it is recognised that people with lower educational or socioeconomic status are less physically active [44]. Similarly, patients who have more active disease, pain or fatigue may be less included to participate in a lifestyle survey. We did not have enough patients with co-morbidities to analyse these separately and it is acknowledged that co-morbid conditions which influence PA [31].

In summary, this study identified variation in PA amongst patients with SLE and the importance of employment in helping to meet international PA targets. Both health beliefs and disease characteristics are associated with engagement in PA. Patients with a more recent diagnosis appear to be more concerned that PA could worsen disease activity or cause injury, which could be addressed by patient education. Opinions surrounding PA differ greatly between patients and so person-specific barriers will need to be addressed.

## Supplementary Information

Below is the link to the electronic supplementary material.ESM 1(PDF 413 KB)ESM 2(DOCX 35.6 KB)

## Data Availability

The data underlying this article will be shared on reasonable request to the corresponding author.

## References

[1] Atzeni F, Rodríguez-Pintó I, Cervera R (2024) Cardiovascular disease risk in systemic lupus erythematous: certainties and controversies. Autoimmun Rev 23:103646 39321952 10.1016/j.autrev.2024.103646

[2] Moustafa AT, Moazzami M, Engel L, Bangert E, Hassanein M, Marzouk S, Kravtsenyuk M, Fung W, Eder L, Su J, Wither JE, Touma Z (2020) Prevalence and metric of depression and anxiety in systemic lupus erythematosus: a systematic review and meta-analysis. Semin Arthritis Rheum 50:84–94 31303437 10.1016/j.semarthrit.2019.06.017

[3] Healey EL, Allen KD, Bennell K, Bowden JL, Quicke JG, Smith R (2020) Self-report measures of physical activity. Arthritis Care Res (Hoboken) 72(Suppl 10):717–730 33091242 10.1002/acr.24211 PMC7586463

[4] Craig CL, Marshall AL, Sjöström M, Bauman AE, Booth ML, Ainsworth BE, Pratt M, Ekelund ULF, Yngve A, Sallis JF, Oja P (2003) International physical activity questionnaire: 12-country reliability and validity. Med Sci Sports Exerc 35:1381–1395 12900694 10.1249/01.MSS.0000078924.61453.FB

[5] Xu B, Ruan P, Wu H, Gao Y, Long H, Wu IXY, Lu Q (2025) Physical activity in relation to the risk of systemic lupus erythematosus: a prospective study including 401 745 individuals. Rheumatology (Oxford) 64:4267–4274 40342027 10.1093/rheumatology/keaf150

[6] Blaess J, Goepfert T, Geneton S, Irenee E, Gerard H, Taesch F, Sordet C, Arnaud L (2023) Benefits & risks of physical activity in patients with systemic lupus erythematosus: a systematic review of the literature. Semin Arthritis Rheum 58:152128 36436314 10.1016/j.semarthrit.2022.152128

[7] O’Dwyer T, Durcan L, Wilson F (2017) Exercise and physical activity in systemic lupus erythematosus: a systematic review with meta-analyses. Semin Arthritis Rheum 47:204–215 28477898 10.1016/j.semarthrit.2017.04.003

[8] Caruso Mazzolani B, Infante Smaira F, Mendes Sieczkowska S, Romero M, Gofinet Pasoto S, de Sá Pinto AL, Rodrigues Lima F, Braga Benatti F, Roschel H, Gualano B (2025) A randomized controlled trial of a lifestyle intervention on wellbeing in patients with systemic lupus erythematosus: results from “Living well with lupus.” Lupus 34:1460–1471 41165066 10.1177/09612033251394423

[9] Zouganeli V, Dimopoulos S, Briasoulis A, Karkamanis A, Panagiotopoulos P, Karatzanos E, Boumpas DT, Vasileiadis I, Nanas S, Kourek C (2025) The effects of exercise training on functional aerobic capacity and quality of life in patients with systemic lupus erythematosus: a systematic review of randomized controlled trials. J Clin Med 14:7031 41096111 10.3390/jcm14197031 PMC12525207

[10] Wohland H, Aringer M, Leuchten N (2024) Physical exercise is associated with less fatigue, less pain and better sleep in patients with systemic erythematosus. Clin Exp Rheumatol 42:601–607 37706288 10.55563/clinexprheumatol/az3pkn

[11] Hasni S, Feng LR, Chapman M, Gupta S, Ahmad A, Munday A, Mazhar MA, Li X, Lu S, Tsai WL, Gadina M, Davis M, Chu J, Manna Z, Nakabo S, Kaplan MJ, Saligan L, Keyser R, Chan L, Chin LMK (2022) Changes in cardiorespiratory function and fatigue following 12 weeks of exercise training in women with systemic lupus erythematosus: a pilot study. Lupus Sci Med 9:e000778 36220328 10.1136/lupus-2022-000778 PMC9557301

[12] Gavilán-Carrera B, Vargas-Hitos JA, Morillas-de-Laguno P, Rosales-Castillo A, Sola-Rodríguez S, Callejas-Rubio JL, Sabio JM, Soriano-Maldonado A (2022) Effects of 12-week aerobic exercise on patient-reported outcomes in women with systemic lupus erythematosus. Disabil Rehabil 44:1863–1871 32878503 10.1080/09638288.2020.1808904

[13] Bogdanovic G, Stojanovich L, Djokovic A, Stanisavljevic N (2015) Physical activity program is helpful for improving quality of life in patients with systemic lupus erythematosus. Tohoku J Exp Med 237:193–199 26490344 10.1620/tjem.237.193

[14] Tench CM, McCarthy J, McCurdie I, White PD, D’Cruz DP (2003) Fatigue in systemic lupus erythematosus: a randomized controlled trial of exercise. Rheumatology (Oxford) 42:1050–1054 12730519 10.1093/rheumatology/keg289

[15] Blaess J, Geneton S, Goepfert T, Appenzeller S, Bordier G, Davergne T, Fuentes Y, Haglo H, Hambly K, Kinnett-Hopkins D, Su K-Y, Legge A, Li L, Mak A, Padjen I, Sciascia S, Sheikh SZ, Soriano-Maldonado A, Ugarte-Gil MF, Md Yusof MY, Parodis I, Arnaud L (2024) Recommendations for physical activity and exercise in persons living with systemic lupus erythematosus (SLE): consensus by an international task force. RMD Open 10:e004171 38580348 10.1136/rmdopen-2024-004171 PMC11002419

[16] Bull FC, Al-Ansari SS, Biddle S, Borodulin K, Buman MP, Cardon G, Carty C, Chaput JP, Chastin S, Chou R, Dempsey PC, DiPietro L, Ekelund U, Firth J, Friedenreich CM, Garcia L, Gichu M, Jago R, Katzmarzyk PT, Lambert E, Leitzmann M, Milton K, Ortega FB, Ranasinghe C, Stamatakis E, Tiedemann A, Troiano RP, van der Ploeg HP, Wari V, Willumsen JF (2020) World Health Organization 2020 guidelines on physical activity and sedentary behaviour. Br J Sports Med 54:1451–1462 33239350 10.1136/bjsports-2020-102955 PMC7719906

[17] Bauman AE, Reis RS, Sallis JF, Wells JC, Loos RJF, Martin BW (2012) Correlates of physical activity: why are some people physically active and others not? Lancet 380:258–271 22818938 10.1016/S0140-6736(12)60735-1

[18] Garcia L, Mendonça G, Benedetti TRB, Borges LJ, Streit IA, Christofoletti M, Silva-Júnior FL, Papini CB, Binotto MA (2022) Barriers and facilitators of domain-specific physical activity: a systematic review of reviews. BMC Public Health 22:1964 36289461 10.1186/s12889-022-14385-1 PMC9598005

[19] Mancuso CA, Perna M, Sargent AB, Salmon JE (2011) Perceptions and measurements of physical activity in patients with systemic lupus erythematosus. Lupus 20:231–242 21183562 10.1177/0961203310383737

[20] Bauman A, Bull F, Chey T, Craig CL, Ainsworth BE, Sallis JF, Bowles HR, Hagstromer H, Sjostrom M, Pratt M (2009) The International Prevalence Study on Physical Activity: results from 20 countries. Int J Behav Nutr Phys Act 6:21 19335883 10.1186/1479-5868-6-21 PMC2674408

[21] Quickfall M, Green S, Hesketh K, Van Zanten JV, Cocks M, Reynolds J, Wadley AJ (2025) EXamining the feasibility of exerCisE to manage symptoms of lupus (EXCEL): a protocol for a randomised controlled pilot study. Lupus Sci Med 12:e001382 39824591 10.1136/lupus-2024-001382 PMC11751982

[22] Sieczkowska SM, Smaira FI, Mazzolani BC, Romero M, Pasoto SG, de Sá Pinto AL, Lima FR, De Oliveira VR, Ueda S, Benatti FB, Roschel H, Gualano B (2023) A randomized controlled trial of an intervention promoting physical activity and healthy eating recommendations in systemic lupus erythematosus: the protocol study “Living Well with Lupus.” Rheumatol Int 43:1799–1810 37354245 10.1007/s00296-023-05370-x

[23] Noble S, McLennan D, Noble M, Plunkett E, Gutacker N, Silk M, Wright G (2019) The English indices of deprivation 2019. Ministry of Housing, communities and local government, London, United Kingdom

[24] Svenungsson E, Gunnarsson I, Illescas-Bäckelin V, Trysberg E, Jönsen A, Leonard D, Sjöwall C, Pettersson S (2021) Quick Systemic Lupus Activity Questionnaire (Q-SLAQ): a simplified version of SLAQ for patient-reported disease activity. Lupus Sci Med 8:e000471 33972457 10.1136/lupus-2020-000471 PMC8112425

[25] Sport E (2025) Active Lives Adult Survey November 2023–24 Report. In:Sport England, London, United Kingdom

[26] Sjostrom M, Ainsworth BE, Bauman A, Bull FC, Hamilton-Craig CR, Sallis JF (2005) Guidelines for data processing analysis of the International Physical Activity Questionnaire (IPAQ) - short and long forms. (https://sites.google.com/view/ipaq/score)

[27] Wl H, Lee I-M, Pate RR, Powell KE, Blair SN, Franklin BA, Macera CA, Heath GW, Thompson PD, Bauman A (2007) Physical Activity and Public Health: updated recommendation for adults from the American College of Sports Medicine and the American Heart Association. Med Sci Sports Exerc 39:1423–1434 17762377 10.1249/mss.0b013e3180616b27

[28] Herrmann SD, Willis EA, Ainsworth BE, Barreira TV, Hastert M, Kracht CL, Schuna JM Jr., Cai Z, Quan M, Tudor-Locke C, Whitt-Glover MC, Jacobs DR Jr. (2024) 2024 Adult compendium of physical activities: a third update of the energy costs of human activities. J Sport Health Sci 13:6–12 38242596 10.1016/j.jshs.2023.10.010 PMC10818145

[29] Sport England. (2025). Active Lives Survey, 2018–2019 (4th ed.) [Data collection]. UK Data Service.

[30] NHS England. Health Survey for England, 2021 [Internet]. Leeds: NHS England; 2022

[31] Cook MJ, Bellou E, Bowes J, Sergeant JC, O’Neill TW, Barton A, Verstappen SMM (2018) The prevalence of co-morbidities and their impact on physical activity in people with inflammatory rheumatic diseases compared with the general population: results from the UK Biobank. Rheumatology (Oxford) 57:2172–2182 30107595 10.1093/rheumatology/key224 PMC6256331

[32] Eriksson K, Svenungsson E, Karreskog H, Gunnarsson I, Gustafsson J, Möller S, Pettersson S, Boström C (2012) Physical activity in patients with systemic lupus erythematosus and matched controls. Scand J Rheumatol 41:290–297 22651371 10.3109/03009742.2011.624117

[33] Ahn GE, Chmiel JS, Dunlop DD, Helenowski IB, Semanik PA, Song J, Ainsworth B, Chang RW, Ramsey-Goldman R (2015) Self-reported and objectively measured physical activity in adults with systemic lupus erythematosus. Arthritis Care Res 67:701–707 10.1002/acr.22480 PMC4370811 25251755

[34] Mischler T, Kawka L, Sarmiento-Monroy JC, Mertz P, Pijnenburg L, Rinagel M, Ugarte-Gil MF, Geneton S, Blaess J, Piga M, Sordet C, Arnaud L (2025) Levels of physical activity in a large international cohort of patients with systemic lupus erythematosus. Lupus Sci Med 12:e001443 40090673 10.1136/lupus-2024-001443 PMC11911810

[35] Beider S, Stephan M, Seeliger T, Skripuletz T, Witte T, Ernst D (2024) A comparative cross-sectional study of psychological distress, fatigue, and physical activity in patients with rheumatoid arthritis, systemic lupus erythematosus, and Sjögren’s disease. Front Med (Lausanne) 11:1507242 39845811 10.3389/fmed.2024.1507242 PMC11752907

[36] Hallal PC, Victora CG, Wells JCK, Lima RC, Valle NJ (2004) Comparison of short and full-length International Physical Activity Questionnaires. J Phys Act Health 1:227–234

[37] Margiotta DPE, Basta F, Dolcini G, Batani V, Lo Vullo M, Vernuccio A, Navarini L, Afeltra A (2018) Physical activity and sedentary behavior in patients with Systemic Lupus Erythematosus. PLoS ONE 13:e0193728 29505598 10.1371/journal.pone.0193728 PMC5837187

[38] Mahieu MA, Ahn GE, Chmiel JS, Dunlop DD, Helenowski IB, Semanik P, Song J, Yount S, Chang RW, Ramsey-Goldman R (2016) Fatigue, patient reported outcomes, and objective measurement of physical activity in systemic lupus erythematosus. Lupus 25:1190–1199 26869353 10.1177/0961203316631632 PMC4980272

[39] Cillekens B, Lang M, van Mechelen W, Verhagen E, Huysmans MA, Holtermann A, van der Beek AJ, Coenen P (2020) How does occupational physical activity influence health? An umbrella review of 23 health outcomes across 158 observational studies. Br J Sports Med 54:1474–1481 33239353 10.1136/bjsports-2020-102587

[40] Cornet A, Andersen J, Myllys K, Edwards A, Arnaud L (2021) Living with systemic lupus erythematosus in 2020: a European patient survey. Lupus Sci Med 8:e000469 33849920 10.1136/lupus-2020-000469 PMC8051432

[41] Smaira FI, Mazzolani BC, Sieczkowska SM, Romero M, Pasoto S, de Sá Pinto AL, Lima FR, Benatti FB, Roschel H, Weber MB, Gualano B (2025) A qualitative analysis of follow-up interviews with SLE patients from the “living well with lupus” study. Front Med (Lausanne) 12:1681780 41179857 10.3389/fmed.2025.1681780 PMC12575327

[42] Barboza JM, Esteves GP, Ribeiro WJ, Fickert VR, Franco AS, Seguro LPC, Roschel H, Gualano B, Dolan E (2025) Barriers and motivations to exercise participation among women with severe systemic lupus erythematosus who have recently undergone glucocorticoid pulse therapy: a qualitative analysis. Lupus 34:492–499 40123164 10.1177/09612033251330116

[43] Morgan C, Bland AR, Maker C, Dunnage J, Bruce IN (2018) Individuals living with lupus: findings from the LUPUS UK members survey 2014. Lupus 27:681–687 29310537 10.1177/0961203317749746 PMC5888773

[44] Farrell L, Hollingsworth B, Propper C, Shields MA (2014) The socioeconomic gradient in physical inactivity: evidence from one million adults in England. Soc Sci Med 123:55–63 25462605 10.1016/j.socscimed.2014.10.039

