# Supplementary material for: Barriers to physical activity in patients with systemic lupus erythematosus in the UK
Source: Clin Rheumatol. 2026 Jul 28;45(9):5481–90. doi: 10.1007/s10067-026-08324-3 (PMC13526127; doi:10.1007/s10067-026-08324-3)
Supplement: Supplementary file 1 [file 10067_2026_8324_MOESM1_ESM.pdf]

UNIVERSITY OF BIRMINGHAM

V2.0: A Lifestyle Survey of Patients with Systemic Lupus Erythematosus (SLE) in the UK

Participant Information Sheet

UNIVERSITY OF BIRMINGHAM

NHS
Sandwell and West Birmingham
NHS Trust

A Lifestyle Survey of Patients with Systemic Lupus Erythematosus (SLE) in the UK

Participant information sheet

Survey, version 2.0 (04-04-2023)

Sponsor study reference: RG_21-080

PIS, version 2.0 (11-11-2022)

Chief Investigator: Dr Alex Wadley

Name of Researchers: Dr John Reynolds, Dr Jet Veldhuijzen van Zanten, Dr Matthew Cocks, Dr Katie Hesketh, Miss Megan Quickfall, Dr Aliaksandra Baranskaya, Mr Scott Green

We invite you to take part in a research study

You are invited to take part in this study to help to better understand the lifestyle of patients with SLE living in the UK. However, before you give consent to participate in the study, it is important that you understand why this research is being completed and what will be required of you. Please take time to read through this information sheet. If there are any areas that are not clear, or that you would like more information on, feel free to contact the researchers who will be happy to provide this information for you.

Why are we doing the study?

SLE is a condition that can affect different people in diverse ways. It is likely that living with a condition like SLE affects your lifestyle in terms of what you are or are not able to do. We think that there are likely to be big differences between individuals and that a "one size fits all" approach to lifestyle interventions is not likely to work. We want to better understand how SLE affects people's lives, so that we can try and improve the day-to-day lives for people with SLE.

What is involved?

If you are happy to take part, we ask that you complete the online consent form on the next page. After that there will be a series of online questions which should take around 25 minutes to complete. Questions are optional and you do not have to answer if you don't want to. Some questions will ask about your personal information such as your age, employment status and postcode. This will provide us with greater insight into understanding you as an individual and the

environmental factors that may be affecting your daily life. All data will be recorded anonymously and processed in a way that ensures that the published results cannot be linked back to you.

What are the benefits of taking part?

There are no direct benefits from taking part in this study. We hope that the findings from this study will help to support future studies of lifestyle interventions in patients with SLE and help the medical community better understand patients with SLE.

Are there any risks if I take part in the study?

The risks involved in the study are low. However, some of the questions in this study ask about your health and well-being, and sometimes people may find this to be a sensitive topic to think about or answer questions on. In view of this we would like to highlight that all of our questions are optional, and if you are finding any of them upsetting, please skip ahead or exit the survey entirely. If you require any further support regarding this, consider contacting your GP or accessing some of the resources mentioned below:

The Samaritans (UK) – general mental health support, including suicidal and other distressing thoughts - https://www.samaritans.org

MIND (UK) – mental health support - https://www.mind.org.uk/

Additionally, the themes explored in the questionnaire may raise questions about your health and well-being. If this happens, we advise that you discuss these matters with your GP or rheumatologist.

Current government guidelines and local safety procedures will be adhered to in relation to the ongoing pandemic in order to minimise risk of exposure to the virus.

What will happen to the data?

The data generated for this study will be securely stored at the University of Birmingham and only accessed by the named researchers. Some of the collected data may be classed as potentially identifiable – which means that if all your answers were put together there is a chance that you as an individual could be identified from them. Only the research team will have access to your data during the analysis stage of the project. Once analysis is complete, the data will be stored in a way that this potentially identifiable data is removed. When the data is published, we will not provide any data on individual participants and so it will not be possible to identify an individual's answers.

How will we use information about you?

We will need to use information from you for this research project.

Only people from the research team will use this information to do the research and all of your information will be kept safe and secure.

Once we have finished the study, we will keep some of the data so we can check the results. We will write our reports in a way that no-one can work out that you took part in the study.

What are your choices about how your information is used?

You can stop being part of the study at any time, without giving a reason, but we will keep information about you that we already have.

We need to manage your records in specific ways for the research to be reliable. This means that we won't be able to let you see or change the data we hold about you.

If you agree to take part in this study, you will have the option to take part in future research using your data saved from this study.

Where can you find out more about how your information is used?

You can find out more about how we use your information

- at www.hra.nhs.uk/information-about-patients/
- by asking one of the research team

- by sending an email to the Chief Investigator: a.j.wadley@bham.ac.uk
- by contacting the University's Data Protection Officer: Dataprotection@contacts.bham.ac.uk

Financial reimbursements

We will not be able to pay participants for their involvement in this project.

What are my rights?

It is your choice whether or not you wish to complete this survey. If you wish to proceed, you will first be given this information sheet to read and be asked to sign a consent form electronically.

Who is funding the study and who has reviewed the study?

This study is sponsored and insured by the University of Birmingham and is funded by the **Lupus UK** (Chief investigator – Dr Alex Wadley). The South East Scotland Research Ethics Committee provided a favourable ethical opinion for this study (IRAS: 311784).

What if I want to make a complaint?

If you wish to complain about any aspect of the way in which you have been approached or treated during the course of this study, you should contact the study investigators who's contact details are available towards the end of this leaflet, in the first instance. If you remain unhappy with their response please contact the Patient Liaison Services (PALS) at your Research Site (Telephone: 0121 507 5836 or email: swb-tr.pals@nhs.net).

I f you have any concerns regarding how your information has been used please contact the University's Data Protection Officer on dataprotection@contacts.bham.ac.uk. If you remain unsatisfied, you may wish to contact the Information Commissioner's Office (ICO). Contact details, and details of data subject rights, are available on the ICO website at: https://ico.org.uk/for-organisations/data-protection-reform/overview-of-the-gdpr/individuals-rights/

What happens now?

You will be asked to complete an informed consent form on the next page to confirm that you are happy to participate in this study. If you agree, the survey will begin on the subsequent page.

Thank you for your time, and if you want to participate in the study or have any further questions, please feel free to contact any of the investigators listed below. If you wish to discuss this with others, contact your GP.

Contact for further information

Thank you for your time reading this document. If there is anything that is not clear, you would like more information or wish to take part in this study, please do not hesitate to contact the research team or chief investigator below:

Research Team

Email: excel-research@contacts.bham.ac.uk

Dr Alex Wadley (Chief Investigator)

Email: a.j.wadley@bham.ac.uk

Consent

Before taking part in this study, I confirm that:

1. I have read and understand the participant information sheet version 2.0 dated 01-11-2022 for the above study. I have had the opportunity to consider the information, ask questions and have had these answered satisfactorily.

2. I understand that my participation is voluntary and that I am free to withdraw at any time without giving any reason, without my medical care or legal rights being affected.

3. I am 18 years or older.

4. I have a diagnosis of systemic lupus erythematosus (SLE)

5. I live in the United Kingdom (UK).

6. I agree to take part in this study.

On this basis, I freely consent to participate in the above study. *NB. Agreement is confirmed by clicking the 'next' button.* ✱ *Required*

○ Yes

Demographics

1. What is your gender?

- ◌ Female
- ◌ Male
- ◌ Prefer not to say

If you are female, are you currently pregnant?

- ◌ Yes
- ◌ No
- ◌ Don't know

How old are you (in years)?

Please enter a whole number (integer).

Please make sure the number is between 18 and 120.

____

How would you describe your ethnicity?

Please select no more than 1 answer(s).

- ☐ Asian (excluding Chinese)
- ☐ Black
- ☐ Chinese
- ☐ Mixed
- ☐ Other Ethnic Group
- ☐ White British
- ☐ White Other
- ☐ Prefer not to answer

Have you been diagnosed to have Systemic Lupus Erythematosus, also known as Lupus or SLE? ✱ *Required*

- ◌ Yes
- ◌ No – please stop at this point as this survey is intended for those with a diagnosis of Lupus.

Lupus Related Questions

For how many years have you had a diagnosis of Lupus? Please round up to the nearest year.

Please enter a whole number (integer).

Did you have symptoms for more than 1 year before a diagnosis of SLE was made? If so, how many years?

- ◯ Yes
- ◯ No, my diagnosis was made within 1 year of symptom onset.

If yes, how many years?

Please enter a whole number (integer).

Which of the following medications are you currently taking specifically for management of your lupus? Select all that apply.

- ☐ Prednisolone or steroid tablets
- ☐ Hydroxychloroquine (or mepacrine)
- ☐ Methotrexate
- ☐ Azathioprine
- ☐ Mycophenolate mofetil (or CellCept or MyFortic)
- ☐ Ciclosporin (or Deximune or Capimune or Neoral)
- ☐ Tacrolimus (or Prograf or Advagraf or Adoport)
- ☐ Leflunomide
- ☐ Other immunosuppressant drug, please specify

Other, please specify

Have you received rituximab in the last 12 months?

- ◯ Yes
- ◯ No
- ◯ Not sure

Have you received cyclophosphamide in the last 12 months?

- ◯ Yes
- ◯ No
- ◯ Not sure

Have you received belimumab in the last 6 weeks?

- Yes
- No
- Not sure

Have you had intravenous steroids (a steroid drip) in the last 6 weeks?

- Yes
- No
- Not sure

Q-SLAQ

Please review the following list of lupus symptoms. IN THE PAST ONE MONTH, how bad has each of the symptoms been?

	Severity			
	Mild	Moderate	Severe	No problem
Lost weight without trying	○	○	○	○
Fatigue	○	○	○	○
Fevers (38.5 degrees C or above) taken by thermometer	○	○	○	○
Swollen glands (nodes) in the neck (lymphadenopathy)	○	○	○	○
Rash on cheeks (shaped like a butterfly)	○	○	○	○
Rash or feeling sick after going out in the sun	○	○	○	○
Sores in the mouth or nose	○	○	○	○
Alopecia (Bald patches on scalp, or clumps of hair on pillow)	○	○	○	○
Shortness of breath	○	○	○	○
Chest pain with deep breath	○	○	○	○
Abdominal pain	○	○	○	○
Forgetfulness	○	○	○	○
Feeling depressed	○	○	○	○
Unusual headaches	○	○	○	○
Muscle weakness	○	○	○	○
Muscle pain	○	○	○	○
Joint pain or stiffness	○	○	○	○
Joint swelling	○	○	○	○

Please rate the disease activity of your lupus DURING THE PAST ONE MONTH on the scale below, where 0 is no activity and 10 is the most activity.

Please don't select more than 1 answer(s) per row.

Please select at least 1 answer(s).

	0	1	2	3	4	5	6	7	8	9	10	
0 No activity	☐	☐	☐	☐	☐	☐	☐	☐	☐	☐	☐	10 Most Activity

Demographics Other

The following questions are to help us understand your environment, health habits and daytime commitments.

Do you currently smoke tobacco?

- ○ Yes
- ○ No

On an average day, how many cigarettes or roll-ups do you smoke?

Please enter a whole number (integer).

[ ]

Do you use electronic cigarettes or vape?

- ○ Yes
- ○ No

Do you drink alcohol?

- ○ Yes
- ○ No

On an average week, how many units of alcohol would you drink? For reference a 175ml glass of 13% wine is 2.3 units, one pint of 4% beer or larger is 2.3 units, one shot of spirits is 1 unit.

Please enter a number.

[ ]

What is your height? Please select a unit, feet and inches or meters, to input your answer.

- ○ Feet and Inches
- ○ Meters

Input height

[ ]

What is your current weight? If you are uncertain, please give an estimate. Please select a unit, stone and pounds or kilograms, to input your answer.

- ○ Stone and pounds
- ○ Kilograms

Input weight

What is your highest educational qualification? This means any educational, professional, vocational or other work-related qualififcation for which you received a certificate?

- Entry Level
- Level 1 - GCSE grades 3, 2, 1 or grades D, E, F, G. Or level 1 certificate/skill/NVQ
- Level 2 - GCSE grades 9, 8, 7, 6, 5, 4 or grades A*, A, B, C, intermediate apprenticeship,, music grades 4 and 5, Level 2 NVQ/Skill/certificate
- Level 3 - AS or A Level, advanced apprenticeship, international Baccalaureate diploma, access to higher education diploma, music grades 6, 7 and 8, Level 3 NVQ/Skill/Certificate
- Level 4 - Certificate of higher education (CertHE), higher apprenticeship, higher national certificate (HNC), Level 4 NVQ/Skill/Certificate
- Level 5 - Diploma of higher education (DipHE), foundation degree, higher national diploma (HND), Level 5 NVQ/Skill/Certificate
- Level 6 - Degree apprenticeship, degree with or without honours, graduate certificate/ diploma, level 6 NVQ/certificate/diploma
- Level 7 -Masters degree, postgraduate certificate/ diploma, postgraduate certificate in education (PGCE), Level 7 NVQ/diploma/certificate
- Level 8 - Doctorate (PhD), Level 8 NVQ/Diploma/Certificate

What is your employment status?

- Working full or part time
- Unemployed
- Not working - retired
- Not working - looking after house/children/ full time carer
- Not working - long term sick or disabled
- Student full or part time
- Other working status

What is your postcode? This is to help us understand how peoples' lifestyles may differ across the UK.

Please enter a valid UK postcode.

Outside of work, do you look after any children under the age of 5? These can be your own children or someone else’s.

- Yes
- No

SRCCI - Comorbidity Questionnaire

The next few questions are about any conditions you may suffer with, except for lupus. They help us get a broader understanding of your physical health.

Have you ever had a heart attack?

- Yes
- No

Have you ever been treated for heart failure? (You may have been short of breath and the doctor may have told you that you had fluid in your lungs or that your heart was not pumping well.)

- Yes
- No

Have you had an operation to unclog or bypass the arteries in your legs?

- Yes
- No

Have you had a stroke, cerebrovascular accident, blood clot or bleeding in the brain, or transient ischemic attack (TIA)?

- Yes
- No

Do you have difficulty moving an arm or leg as a result of the stroke or cerebrovascular accident?

- Yes
- No

Do you have asthma? If yes, do you take medicines for your asthma?

- No
- Yes, only with flare-ups of my asthma
- Yes, I take medicines regularly, even when I'm not having a flare-up.

Do you have emphysema, chronic bronchitis, or chronic obstructive lung disease? If yes, do you take medicines for your lung disease?

- No
- Yes, only with flare-ups of my asthma

- Yes, I take medicines regularly, even when I'm not having a flare-up.

Do you have stomach ulcers, or peptic ulcer disease?

- Yes
- No

If yes, has this condition been diagnosed by endoscopy (where a doctor looks into your stomach through a scope) or an upper gi or barium swallow study (where you swallow chalky dye and then xrays are taken)?

- Yes
- No

Do you have diabetes (high blood sugar)?

- No
- Yes, treated by modifying my diet
- Yes, treated by medications taken by mouth
- Yes, treated by insulin injections

Has the diabetes caused any of the following problems?

- [ ] Problems with your kidneys
- [ ] Problems with your eyes, treated by an ophthalmologist

Have you ever had the following problems with your kidneys? Select those that apply

- [ ] Poor kidney function (blood tests show high creatinine)
- [ ] Have used hemodialysis or peritoneal dialysis
- [ ] Have received kidney transplantation

Do you have rheumatoid arthritis?

- Yes
- No

Do you take medications for it regularly?

- Yes
- No

Do you have Polymalgia rheumatica?

- Yes
- No

Do you have any of the following conditions? Select all that apply.

- Alzheimer's Disease, or another form of dementia
- Cirrhosis, or serious liver damage
- Leukemia or polycythemia vera
- Lymphoma
- Cancer, other than skin cancer, leukemia or lymphoma
- AIDS

Cancer, other than skin cancer, leukemia or lymphoma. If yes, has the cancer spread, or metastasized to other parts of your body?

- yes
- no
- don't know

IPAQ

We are interested in finding out about the kinds of physical activities that people do as part of their everyday lives. The questions will ask you about the time you spent being physically active in the **last 7 days**. Please answer each question even if you do not consider yourself to be an active person. Please think about the activities you do at work, as part of your house and yard (garden) work, to get from place to place, and in your spare time for recreation, exercise or sport.

Think about all the **vigorous** and **moderate** activities that you did in the **last 7 days. Vigorous** physical activities refer to activities that take hard physical effort and make you breathe much harder than normal. **Moderate** activities refer to activities that take moderate physical effort and make you breathe somewhat harder than normal.

PART 1: JOB-RELATED PHYSICAL ACTIVITY

The first section is about your work. This includes paid jobs, farming, volunteer work, course work, and any other unpaid work that you did outside your home. Do not include unpaid work you might do around your home, like housework, yard (garden) work, general maintenance, and caring for your family. These are asked in Part 3.

Do you currently have a job or do any unpaid work outside your home?

- ○ Yes
- ○ No

IPAQ - PART 1

The next questions are about all the physical activity you did in the **last 7 days** as part of your paid or unpaid work. This does not include traveling to and from work.

During the **last 7 days**, on how many days did you do **vigorous** physical activities like heavy lifting, digging, heavy construction, or climbing up stairs **as part of your work**? Think about only those physical activities that you did for at least 10 minutes at a time.

How much time did you usually spend on one of those days doing **vigorous** physical activities as part of your work? Please input your answer in **hours** and **minutes per day**. *For example, 1 hour and 20 minutes would be written as 01:20, and 15 minutes would be written as 00:15.*

(hh:mm)

Again, think about only those physical activities that you did for at least 10 minutes at a time. During the **last 7 days**, on how many days did you do **moderate** physical activities like carrying light loads **as part of your work**? Please do not include walking.

How much time did you usually spend on one of those days doing **moderate** physical activities as part of your work? Please input your answer in **hours** and **minutes per day**. *For example, 1 hour and 20 minutes would be written as 01:20, and 15 minutes would be written as 00:15.*

(hh:mm)

During the **last 7 days**, on how many days did you **walk** for at least 10 minutes at a time **as part of your work**? Please do not count any walking you did to travel to or from work.

How much time did you usually spend on one of those days **walking** as part of your work? Please input your answer in **hours** and **minutes per day**. *For example, 1 hour and 20 minutes would be written as 01:20, and 15 minutes would be written as 00:15.*

(hh:mm)

IPAQ - PART 2

PART 2: TRANSPORTATION PHYSICAL ACTIVITY

These questions are about how you travelled from place to place, including to places like work, stores, movies, and so on.

During the **last 7 days**, on how many days did you **travel in a motor vehicle** like a train, bus, car, or tram?

How much time did you usually spend on one of those days **traveling** in a train, bus, car, tram, or other kind of motor vehicle? Please input your answer in **hours** and **minutes per day**. *For example, 1 hour and 20 minutes would be written as 01:20, and 15 minutes would be written as 00:15.*

(hh:mm)

Now think only about the **bicycling** and **walking** you might have done to travel to and from work, to do errands, or to go from place to place.

During the **last 7 days**, on how many days did you **bicycle** for at least 10 minutes at a time to go **from place to place?**

How much time did you usually spend on one of those days to **bicycle** from place to place? Please input your answer in **hours** and **minutes per day**. *For example, 1 hour and 20 minutes would be written as 01:20, and 15 minutes would be written as 00:15.*

(hh:mm)

During the **last 7 days**, on how many days did you **walk** for at least 10 minutes at a time to go **from place to place**?

How much time did you usually spend on one of those days **walking** from place to place? Please input your answer in **hours** and **minutes per day**. *For example, 1 hour and 20 minutes would be written as 01:20, and 15 minutes would be written as 00:15.*

(hh:mm)

IPAQ - PART 3

PART 3: HOUSEWORK, HOUSE MAINTENANCE, AND CARING FOR FAMILY

This section is about some of the physical activities you might have done in **the last 7 days** in and around your home, like housework, gardening, yard work, general maintenance work, and caring for your family.

Think about only those physical activities that you did for at least 10 minutes at a time. During the **last 7 days**, on how many days did you do **vigorous** physical activities like heavy lifting, chopping wood, shovelling snow, or digging **in the garden or yard**?

How much time did you usually spend on one of those days doing **vigorous** physical activities in the garden or yard? Please input your answer in **hours** and **minutes per day**. *For example, 1 hour and 20 minutes would be written as 01:20, and 15 minutes would be written as 00:15.*

(hh:mm)

Again, think about only those physical activities that you did for at least 10 minutes at a time. During the **last 7 days**, on how many days did you do **moderate** activities like carrying light loads, sweeping, washing windows, and raking **in the garden or yard**?

How much time did you usually spend on one of those days doing **moderate** physical activities in the garden or yard? Please input your answer in **hours** and **minutes per day**. *For example, 1 hour and 20 minutes would be written as 01:20, and 15 minutes would be written as 00:15.*

(hh:mm)

Once again, think about only those physical activities that you did for at least 10 minutes at a time. During the **last 7 days**, on how many days did you do moderate activities like carrying light loads, washing windows, scrubbing floors and sweeping **inside your home**?

How much time did you usually spend on one of those days doing **moderate** physical activities inside your home? Please input your answer in **hours** and **minutes per day**. *For example, 1 hour and 20 minutes would be written as 01:20, and 15 minutes would be written as 00:15.*

(hh:mm)

IPAQ - PART 4

PART 4: RECREATION, SPORT, AND LEISURE-TIME PHYSICAL ACTIVITY

This section is about all the physical activities that you did in the **last 7 days** solely for recreation, sport, exercise or leisure. Please do not include any activities you have already mentioned.

Not counting any walking you have already mentioned, during the **last 7 days,** on how many days did you **walk** for at least 10 minutes at a time **in your leisure time**?

How much time did you usually spend on one of those days **walking** in your leisure time? Please input your answer in **hours** and **minutes per day**. *For example, 1 hour and 20 minutes would be written as 01:20, and 15 minutes would be written as 00:15.*

(hh:mm)

Think about only those physical activities that you did for at least 10 minutes at a time. During the **last 7 days**, on how many days did you do **vigorous** physical activities like aerobics, running, fast bicycling, or fast swimming **in your leisure time**?

How much time did you usually spend on one of those days doing **vigorous** physical activities in your leisure time? Please input your answer in **hours** and **minutes per day**. *For example, 1 hour and 20 minutes would be written as 01:20, and 15 minutes would be written as 00:15.*

(hh:mm)

Again, think about only those physical activities that you did for at least 10 minutes at a time. During the **last 7 days**, on how many days did you do **moderate** physical activities like bicycling at a regular pace, swimming at a regular pace, and doubles tennis **in your leisure time?**

How much time did you usually spend on one of those days doing **moderate** physical activities in your leisure time? Please input your answer in **hours** and **minutes per day**. *For example, 1 hour and 20 minutes would be written as 01:20, and 15 minutes would be written as 00:15.*

(hh:mm)

IPAQ - PART 5

PART 5: TIME SPENT SITTING

The last questions are about the time you spend sitting while at work, at home, while doing course work and during leisure time. This may include time spent sitting at a desk, visiting friends, reading or sitting or lying down to watch television. Do not include any time spent sitting in a motor vehicle that you have already told me about.

During the **last 7 days**, how much time did you usually spend **sitting** on a **weekday**? Please input your answer in **hours** and **minutes per day**. *For example, 1 hour and 20 minutes would be written as 01:20, and 15 minutes would be written as 00:15.*

Times need to be in the format 'HH:MM', for example 15:43.

(hh:mm)

During the **last 7 days**, how much time did you usually spend **sitting** on a **weekend day**? Please input your answer in **hours** and **minutes per day**. *For example, 1 hour and 20 minutes would be written as 01:20, and 15 minutes would be written as 00:15.*

(hh:mm)

Leisure Time Activity

Over next few questions we are interested in finding out more about the physical activities that you do **solely for recreation, sport, exercise or leisure**. Please only think about the activity you have participated in over the **last 4 weeks**.

In the **last 4 weeks**, have you done any **Creative or artistic dance**?

More info

- Yes
- No

During the past 4 weeks, on how many days did you do creative or artistic dance?

Please enter a whole number (integer).

Please make sure the number is between 0 and 28.

How much time did you usually spend doing creative or artistic dance on each day that you did the activity? Please input your answer in **hours** and **minutes per day**. *For example, 1 hour and 20 minutes would be written as 01:20, and 15 minutes would be written as 00:15.*

(hh:mm)

Was the effort you put into the activity usually enough to **raise your breathing rate**?

- Yes
- No

Was the effort you put into the activity usually enough to **make you sweat or out of breath?**

- Yes
- No

In the **last 4 weeks**, have you done any **Cycling**?

More info

- Yes
- No

During the past 4 weeks, on how many days did you do cycling?

Please enter a whole number (integer).

Please make sure the number is between 0 and 28.

How much time did you usually spend cycling on each day that you did the activity? Please input your answer in **hours** and **minutes per day**. *For example, 1 hour and 20 minutes would be written as 01:20, and 15 minutes would be written as 00:15.*

(hh:mm)

Was the effort you put into the activity usually enough to **raise your breathing rate**?

- Yes
- No

Was the effort you put into the activity usually enough to **make you sweat or out of breath**?

- Yes
- No

In the **last 4 weeks**, have you done any **Fitness activities**?

More info

- Yes
- No

During the past 4 weeks, on how many days did you do Fitness activities?

Please enter a whole number (integer).

Please make sure the number is between 0 and 28.

How much time did you usually spend doing Fitness activities on each day that you did the activity? Please input your answer in **hours** and **minutes per day**. *For example, 1 hour and 20 minutes would be written as 01:20, and 15 minutes would be written as 00:15.*

(hh:mm)

Was the effort you put into the activity usually enough to **raise your breathing rate**?

- Yes
- No

Was the effort you put into the activity usually enough to **make you sweat or out of breath?**

- Yes
- No

In the **last 4 weeks**, have you done any **Sporting activities**?

More info

- Yes
- No

During the past 4 weeks, on how many days did you do the sporting activities?

Please enter a whole number (integer).

Please make sure the number is between 0 and 28.

[ ]

How much time did you usually spend doing sporting activities on each day that you did the activity? Please input your answer in **hours** and **minutes per day**. *For example, 1 hour and 20 minutes would be written as 01:20, and 15 minutes would be written as 00:15.*

[ ]
(hh:mm)

Was the effort you put into the activity usually enough to **raise your breathing rate**?

- ○ Yes
- ○ No

Was the effort you put into the activity usually enough to **make you sweat or out of breath?**

- ○ Yes
- ○ No

What kind of sporting activities have you carried out? **Select all that apply**.

- ☐ Adventure sports – hiking, hill or mountain walking, rock climbing and etc
- ☐ Bowls
- ☐ Combat sports, martial arts or target sports
- ☐ Gymnastics, trampolining or cheerleading
- ☐ Leisure time games and activities
- ☐ Racket sports
- ☐ Roller or skating sports
- ☐ Running, athletics or multi-sports
- ☐ Swimming
- ☐ Team sports
- ☐ Water sports – swimming and other
- ☐ Winter sports
- ☐ Other

If you selected Other, please specify:

[ ]

In the **last 4 weeks**, have you done any **Walking**? This includes dog walking, rambling, and Nordic walking, as well as walking for travel. **Do not** count time spent walking around shops.

- ○ Yes
- ○ No

During the past 4 weeks, on how many days did you do the walking activity?

Please enter a whole number (integer).

Please make sure the number is between 0 and 28.

How much time did you usually spend walking on each day that you did the activity? Please input your answer in **hours** and **minutes per day**. *For example, 1 hour and 20 minutes would be written as 01:20, and 15 minutes would be written as 00:15.*

(hh:mm)

Was the effort you put into the activity usually enough to **raise your breathing rate**?

- Yes
- No

Was the effort you put into the activity usually enough to **make you sweat or out of breath?**

- Yes
- No

Perceptions

The next few questions are about your personal views on exercise.

How satisfied are you with the amount of leisure time physical activity / exercise you carry out? Please mark on a scale where 0 represents very unsatisfied, wish to do more and 10 represents very satisfied.

Please don't select more than 1 answer(s) per row.

Please select at least 1 answer(s).

	0	1	2	3	4	5	6	7	8	9	10	
0 Very Unsatisfied, wish to do more	☐	☐	☐	☐	☐	☐	☐	☐	☐	☐	☐	10 Very Satisfied

Which setting do you prefer to carry out your leisure time physical activity?

- ○ At home
- ○ Indoor facility such as gym or fitness centre
- ○ Outside
- ○ No preference

Which type of exercise / physical activity you find the **most** enjoyable?

- ○ Group classes
- ○ Group sports
- ○ Visually lead activity at home – such as a workout DVD, youtube video
- ○ Structured Independent activity – this would be activity you do independently but with an outlined plan that you follow such from a written workout plan/ training regime.
- ○ Non structured independent activity
- ○ Non structured activity with a companion – such as going for hike with a friend
- ○ I don’t enjoy any type of exercise or physical activity
- ○ Other

If you selected Other, please specify:

[ ]

What impact do you think regular exercise and physical activity has on your lupus symptoms? Please mark on a scale where you think it reflects your view, 0 is significant worsening of symptoms, 10 is significant improvement of symptoms.

Please don't select more than 1 answer(s) per row.

Please select at least 1 answer(s).

	0	1	2	3	4	5	6	7	8	9	10	

0 significant worsening of symptoms	☐	☐	☐	☐	☐	☐	☐	☐	☐	☐	☐	10 significant improvement of symptoms

What prevents you from carrying out more leisure time exercise / physical activity? **Select all that apply**

- ☐ Caregiving
- ☐ Concern that injury may occur
- ☐ Concern that lupus may get worse
- ☐ Dislike of exercise / no enjoyable form of exercise
- ☐ Fatigue
- ☐ Financial strains
- ☐ Lupus Flares or lupus related symptoms
- ☐ Illnesses other than lupus and their symptoms
- ☐ Lack of access to facilities – gym etc
- ☐ Lack of knowledge on which exercise to do
- ☐ Lack of motivation
- ☐ Lack of time
- ☐ Pain
- ☐ Self-consciousness
- ☐ Weather
- ☐ Other

If you selected Other, please specify:

____

If you had to pick only one factor, which **one** of the factors **prevents you most?**

____

If you selected Other, please specify:

____

Electronic Fitness Devices and PT

Next few questions are about your personal use of electronic fitness devices and Personal Training (PT) sessions.

Do you track your daily physical activity with a device such as a fitness tracker, FIT bit, smart phone, apple watch or etc?

- Yes
- No

Do you have a daily step count goal?

- Yes
- No

What is your daily step count goal?

Please enter a whole number (integer).

steps

How often do you meet your daily step goal?

- Daily
- Few times a week
- Once a week
- Less than once a week
- Once a month
- Less than once a month

Have you ever had personal training sessions / PT sessions ?

- Yes
- No
- Not sure

What was your experience of PT sessions? Please mark your experience on a scale where 0 represent very unsatisfactory and 10 represents very satisfactory

Please don't select more than 1 answer(s) per row.

Please select at least 1 answer(s).

	0	1	2	3	4	5	6	7	8	9	10	
0 Very unsatisfactory	☐	☐	☐	☐	☐	☐	☐	☐	☐	☐	☐	10 Very satisfactory

HAQ

Next set of questions are about your health-related quality of life. Please try to answer each question, even if you do not think it is related to you at this time. There are no right or wrong answers. Please answer exactly as you think or feel. Thank-you.

Please select the **ONE** best answer for your abilities **OVER THE PAST WEEK**:

DRESSING & GROOMING

	Without ANY difficulty	With SOME difficulty	With MUCH difficulty	UNABLE to do
Dress yourself, including tying shoelaces and doing buttons?	○	○	○	○
Shampoo your hair?	○	○	○	○

ARISING

	Without ANY difficulty	With SOME difficulty	With MUCH difficulty	UNABLE to do
Stand up from an armless chair?	○	○	○	○
Get in and out of bed?	○	○	○	○

EATING

	Without ANY difficulty	With SOME difficulty	With MUCH difficulty	UNABLE to do
Cut your meat?	○	○	○	○
Lift a full cup or glass to your mouth?	○	○	○	○
Open a new milk carton?	○	○	○	○

WALKING

	Without ANY difficulty	With SOME difficulty	With MUCH difficulty	UNABLE to do
Walk outdoors on flat ground?	○	○	○	○
Climb five steps?	○	○	○	○

Please select any **AIDS OR DEVICES** that you usually use for any of the above activities:

- ☐ Cane
- ☐ Walker
- ☐ Crutches
- ☐ Wheelchair
- ☐ Devices used for dressing (button hook, zipper puller, etc)
- ☐ Built-up or special utensils
- ☐ Special or built up chair
- ☐ Other

If you selected Other, please specify:

Please select any categories for which you need **HELP FROM ANOTHER PERSON**

- ☐ Dressing and Grooming
- ☐ Arising
- ☐ Eating
- ☐ Walking

HAQ Continued

OVER THE PAST WEEK were you able to:

HYGEINE

	Without ANY difficulty	With SOME difficulty	With MUCH difficulty	UNABLE to do
Wash and dry your entire body?	○	○	○	○
Take a tub bath?	○	○	○	○
Get on and off the toilet?	○	○	○	○

REACH

	Without ANY difficulty	With SOME difficulty	With MUCH difficulty	UNABLE to do
Reach and get a 5-lb object (such as a filled 2 litre bottle of water) from just above your head?	○	○	○	○
Bend down and pick up clothing from the floor?	○	○	○	○

GRIP

	Without ANY difficulty	With SOME difficulty	With MUCH difficulty	UNABLE to do
Open car doors?	○	○	○	○
Open jars which have been previously opened?	○	○	○	○
Turn faucets (taps) on and off?	○	○	○	○

ACTIVITIES

	Without ANY difficulty	With SOME difficulty	With MUCH difficulty	UNABLE to do
Run errands and shop?	○	○	○	○
Get in and out of a car?	○	○	○	○
Do chores such as vacuuming, yard (garden) work?	○	○	○	○

Please check any AIDS OR DEVICES that you usually use for any of the above activities:

- ☐ Raised toilet seat
- ☐ Bathtub seat
- ☐ Jar opener
- ☐ Long-handled appliances for reach
- ☐ Long-handled appliances in bathroom
- ☐ Bathtub bar
- ☐ Other

If you selected Other, please specify:

Please check any categories for which you NEED HELP FROM ANOTHER PERSON

- ☐ Hygiene
- ☐ Reach
- ☐ Gripping and opening things
- ☐ Errands and chores

VAS

How much **PAIN** have you had because of your illness in the **PAST WEEK**? Please indicate on the scale below how severe your pain has been:

Please don't select more than 1 answer(s) per row.

Please select at least 1 answer(s).

	0	1	2	3	4	5	6	7	8	9	10	
NO PAIN 0	☐	☐	☐	☐	☐	☐	☐	☐	☐	☐	☐	VERY SEVERE PAIN 10

How much of a problem has **UNUSUAL** fatigue or tiredness been for you **OVER THE PAST WEEK**?

Please don't select more than 1 answer(s) per row.

Please select at least 1 answer(s).

	0	1	2	3	4	5	6	7	8	9	10	
FATIGUE IS NO PROBLEM 0	☐	☐	☐	☐	☐	☐	☐	☐	☐	☐	☐	FATIGUE IS A MAJOR PROBLEM 10

How much of a problem has **SLEEPING** been for you **OVER THE PAST WEEK**?

Please don't select more than 1 answer(s) per row.

Please select at least 1 answer(s).

	0	1	2	3	4	5	6	7	8	9	10	
SLEEP IS NO PROBLEM 0	☐	☐	☐	☐	☐	☐	☐	☐	☐	☐	☐	SLEEP IS A MAJOR PROBLEM 10

Overall, how has your **MOOD** been **OVER THE PAST WEEK**?

Please don't select more than 1 answer(s) per row.

Please select at least 1 answer(s).

	0	1	2	3	4	5	6	7	8	9	10	
VERY LOW 0	☐	☐	☐	☐	☐	☐	☐	☐	☐	☐	☐	VERY HAPPY 10

Muscle Strength Questions

Do you currently feel you have a reduction in:

	A lot	Some	A little	None
The strength in your arms?	○	○	○	○
The strength in your legs?	○	○	○	○
Your muscle mass?	○	○	○	○
Your energy?	○	○	○	○
Your physical capabilities?	○	○	○	○
Your general flexibility?	○	○	○	○

Do you have pain in your muscles?

- Often
- Sometimes
- Rarely
- Never

Do you feel physically weak?

- Yes, completely
- Yes, somewhat
- Yes, a little
- No, not at all

Do you have problems with your balance?

- Often
- Occasionally
- Rarely
- Never

How often do you fall?

- Very often
- Occasionally
- Rarely
- Never

Does your muscle weakness limit your movement?

- Yes, a lot

- Yes, somewhat
- Yes, a little
- No, not at all

If yes, for what reasons? Choose as many answers as you like.

If you selected Other, please specify:

Final page

End of questionnaire.

Thank you for your participation.

Key for selection options

38 - During the last 7 days, on how many days did you do vigorous physical activities like heavy lifting, digging, heavy construction, or climbing up stairs as part of your work? Think about only those physical activities that you did for at least 10 minutes at a time.

No vigorous job-related physical activity
One day per week
Two days per week
Three days per week
Four days per week
Five days per week
Six days per week
Seven days per week

39 - Again, think about only those physical activities that you did for at least 10 minutes at a time. During the last 7 days, on how many days did you do moderate physical activities like carrying light loads as part of your work? Please do not include walking.

No moderate job-related physical activity
One day per week
Two days per week
Three days per week
Four days per week
Five days per week
Six days per week
Seven days per week

40 - During the last 7 days, on how many days did you walk for at least 10 minutes at a time as part of your work? Please do not count any walking you did to travel to or from work.

No job-related walking
One day a week
Two days a week
Three days a week
Four days a week
Five days a week
Six days a week
Seven days a week

41 - During the last 7 days, on how many days did you travel in a motor vehicle like a train, bus, car, or tram?

No traveling in a motor vehicle
One day a week
Two days a week
Three days a week
Four days a week
Five days a week
Six days a week
Seven days a week

42 - During the last 7 days, on how many days did you bicycle for at least 10 minutes at a time to go from place to place?

No bicycling from place to place
One day a week
Two days a week
Three days a week
Four days a week
Five days a week
Six days a week
Seven days a week

43 - During the last 7 days, on how many days did you walk for at least 10 minutes at a time to go from place to place?

No walking from place to place
One day a week
Two days a week
Three days a week
Four days a week
Five days a week
Six days a week
Seven days a week

44 - Think about only those physical activities that you did for at least 10 minutes at a time. During the last 7 days, on how many days did you do vigorous physical activities like heavy lifting, chopping wood, shovelling snow, or digging in the garden or yard?

No vigorous activity in garden or yard
One day a week
Two days a week
Three days a week
Four days a week
Five days a week
Six days a week
Seven days a week

45 - Again, think about only those physical activities that you did for at least 10 minutes at a time. During the last 7 days, on how many days did you do moderate activities like carrying light loads, sweeping, washing windows, and raking in the garden or yard?

No moderate activity in garden or yard
One day a week
Two days a week
Three days a week
Four days a week
Five days a week
Six days a week
Seven days a week

46 - Once again, think about only those physical activities that you did for at least 10 minutes at a time. During the last 7 days, on how many days did you do moderate activities like carrying light loads, washing windows, scrubbing floors and sweeping inside your home?

No moderate activity inside home
One day a week
Two days a week
Three days a week
Four days a week
Five days a week
Six days a week
Seven days a week

47 - Not counting any walking you have already mentioned, during the last 7 days, on how many days did you walk for at least 10 minutes at a time in your leisure time?

No walking in leisure time
One day a week
Two days a week
Three days a week
Four days a week
Five days a week
Six days a week
Seven days a week

48 - Think about only those physical activities that you did for at least 10 minutes at a time. During the last 7 days, on how many days did you do vigorous physical activities like aerobics, running, fast bicycling, or fast swimming in your leisure time?

No vigorous activity in leisure time
One day a week
Two days a week
Three days a week
Four days a week
Five days a week

Six days a week
Seven days a week

49 - Again, think about only those physical activities that you did for at least 10 minutes at a time. During the last 7 days, on how many days did you do moderate physical activities like bicycling at a regular pace, swimming at a regular pace, and doubles tennis in your leisure time?

No moderate activity in leisure time
One day a week
Two days a week
Three days a week
Four days a week
Five days a week
Six days a week
Seven days a week

62 - If you had to pick only one factor, which one of the factors prevents you most?

Caregiving
Concern that injury may occur
Concern that lupus may get worse
Dislike of exercise / no enjoyable form of exercise
Fatigue
Financial strains
Lupus Flares or lupus related symptoms
Illnesses other than lupus and their symptoms
Lack of access to facilities – gym etc
Lack of knowledge on which exercise to do
Lack of motivation
Lack of time
Pain
Self-consciousness
Weather
Other

86.a - If yes, for what reasons? Choose as many answers as you like.

Fear of pain
Fear that you might not be able to
Fear of feeling tired after these activities
Fear of falling
Other
