# Supplementary material for: Barriers to physical activity in patients with systemic lupus erythematosus in the UK
Source: Clin Rheumatol. 2026 Jul 28;45(9):5481–90. doi: 10.1007/s10067-026-08324-3 (PMC13526127; doi:10.1007/s10067-026-08324-3)
Supplement: Supplementary file 2 [file 10067_2026_8324_MOESM2_ESM.docx]

**Supplementary file 2:**

***Supplementary Table S1:*** Current medication use in the 268 patients with SLE

|  | N (%) |
| --- | --- |
| Prednisolone | 87 (32.5%) |
| Hydroxychloroquine | 216 (80.6%) |
| Methotrexate | 41 (15.3%) |
| Azathioprine | 29 (10.8%) |
| Mycophenolate mofetil | 57 (21.3%) |
| Ciclosporin | 1 (0.37%) |
| Tacrolimus | 4 (1.49%) |
| Leflunomide | 3 (1.12%) |
| Other | 5 (1.87%) |
| Rituximab in the last 12 months | 15 (5.64%) |
| Cyclophosphamide in the last 12 months | 4 (1.15%) |
| Belimumab in the last 6 weeks | 6 (2.26%) |
| Intravenous steroids in the last 6 weeks | 8 (3.01%) |

***Supplementary Table S2:*** Comorbidities in the 268 patients with SLE

|  | N (%) |
| --- | --- |
| Cardiovascular | |
| Myocardial infarction | 6 (2.26%) |
| Heart failure | 14 (5.32%) |
| Peripheral vascular disease | 1 (0.38%) |
| Stroke or TIA | 24 (9.09%) |
| Respiratory | |
| Asthma | 72 (27.2%) |
| COPD | 10 (3.83%) |
| Other | |
| Peptic ulcers | 17 (6.51%) |
| Diabetes | 6 (2.26%) |
| Kidney disease | 79 (29.5%) |
| Renal transplant | 0 |
| Rheumatoid arthritis | 61 (23.4%) |
| Polymyalgia rheumatica | 9 (3.50%) |
| Dementia | 0 |
| Serious liver disease | 1 (0.37%) |
| Leukaemia | 0 |
| Lymphoma | 2 (0.75%) |
| Other cancer | 3 (1.12%) |
| HIV/AIDS | 0 |

***Supplementary Table S3:*** Characteristics of the 40 patients excluded from IPAQ analysis

|  | | **Total respondents**  **(n=268)** | **Excluded for any reason**  **(n=40)** | **Excluded due to age > 69 years**  **(n=22)** |
| --- | --- | --- | --- | --- |
| Sex: Female (n=267) | | 258 (96.3%) | 37 (92.5%) | 19 (86.4%) |
| Age (years) | | 51 (39-59) | 70 (55-73) | 72 (71-75) |
| Ethnicity | | |  |  |
|  | White | 229 (85.4%) | 33 (82.5%) | 20 (90.9%) |
|  | Asian (including Chinese) | 11 (4.1%) | 2 (5.0%) | 1 (4.55%) |
|  | Black | 9 (3.4%) | 1 (2.5%) | 0 |
|  | Mixed | 12 (4.5%) | 2 (5.0%) | 1 (4.55%) |
|  | Other Ethnic Group | 7 (2.6%) | 2 (5.0%) | 0 |
| UK Country (from postcode) (n=256) | |  |  |  |
|  | England | 216 (84.4%) | 31 (83.8%) | 17 (84.4%) |
|  | Scotland | 19 (7.4%) | 3 (8.1%) | 19 (7.4%) |
|  | Wales | 16 (6.3%) | 3 (8.1%) | 16 (6.3%) |
|  | Northern Ireland | 5 (2.0%) | 0 | 0 |
| Socioeconomic Deprivation based on English IMD 2019 (n=214) | | |  |  |
|  | IMD quintile 1 (most deprived) | 20 (9.3%) | 0 | 0 |
|  | IMD quintile 2 | 43 (20.1%) | 7 (22.6%) | 3 (17.7%) |
|  | IMD quintile 3 | 41 (19.2%) | 6 (16.1%) | 5 (29.4%) |
|  | IMD quintile 4 | 63 (29.4%) | 15 (48.4%) | 9 (52.9%) |
|  | IMD quintile 5 (least deprived) | 47 (22.0%) | 4 (12.9%) | 0 |
| Lupus disease features | |  |  |  |
| Disease duration (years) (n=263) | | 10 (4-20) | 11.5 (7-26) | 20 (8-35) |
| Total Q-SLAQ score (n=243) | | 14 (10-19) | 14 (7-22) | 12 (7-21) |
| Self-reported lupus disease activity (0-10) (n=263) | | 5 (2-7) | 4.5 (2-6.5) | 3.5 (1-6) |
| Lifestyle | |  |  |  |
| BMI (kg/m^2^): (n=261) | | 27.1 (23.5-32.1) | 25.8 (23.4-31.1) | 25.8 (22.5-33.8) |
| Smoking status (n=265) | |  |  |  |
|  | Current tobacco smoker | 12 (4.5%) | 1 (2.6%) | 0 |
|  | Current e-cigarette/vape smoker  Smokes both tobacco and e-cigarette/vape | 12 (4.5%)  2 (0.8%) | 1 (2.6%)  0 | 1 (2.6%)  0 |
| Alcohol consumption (Yes): (n=267) | | 130 (48.7%) | 17 (43.6%) | 7 (31.8%) |
| Units per week (in those answering yes to above) (n=125) | | 5 (2.3-8.0) | 5 (2-11) | 11 (2-14) |
| Employment status (n=264) | |  |  |  |
|  | Working (full or part time) | 145 (54.9%) | 13 (33.3%) | 1 (4.76%) |
|  | Unemployed | 4 (1.5%) | 0 | 0 |
|  | Retired | 46 (17.4%) | 17 (43.6%) | 16 (76.2%) |
|  | Sick/disabled | 46 (17.4%) | 6 (15.4%) | 2 (9.5%) |
|  | Carer/homemaker | 11 (4.2%) | 1 (2.6%) | 0 |
|  | Student | 7 (2.7%) | 0 | 0 |
|  | Other | 5 (1.9%) | 2 (5.1%) | 2 (9.5%) |
| Highest educational attainment (n=259) | |  |  |  |
|  | No secondary education qualification | 11 (4.2%) | 4 (10.8%) | 3 (15%) |
|  | Secondary education | 28 (10.8%) | 6 (16.2%) | 3 (15%) |
|  | Higher secondary | 51 (19.7%) | 6 (16.2%) | 3 (15%) |
|  | Degree or equivalent or above | 169 (65.3%) | 21 (56.8%) | 11 (55%) |

***Values are* median (IQR) or n (%)**

***Supplementary Table S4:*** Preferred type of exercise or physical activity (most enjoyable) (n=266)

|  | N (%) |
| --- | --- |
| Group classes | 42 (15.8%) |
| Group sports | 5 (1.88%) |
| Visually led at home (e.g. DVD) | 30 (11.3%) |
| Structured independent | 47 (17.7%) |
| Non-structured independent | 63 (23.7%) |
| Non-structured with companion | 54 (20.3%) |
| None | 16 (6.02%) |
| Other | 9 (3.38%) |

***Supplementary Table S5***: Preferred type of sports

|  | N (%) |
| --- | --- |
| Adventure | 9/286 (3.36%) |
| Bowls | 0 |
| Combat sports | 1/268 (0.37%) |
| Gymnastics | 2/268 (0.75%) |
| Leisure time games and activities | 1/268 (0.37%) |
| Racket sports | 6/268 (2.24%) |
| Roller or skating sports | 0 |
| Running, athletics or multi-sports | 10/268 (3.73%) |
| Swimming | 11/268 (4.10%) |
| Team sports | 2/268 (0.75%) |
| Water sports | 6/268 (2.24%) |
| Winter sports | 0 |
| Other* | 17/268 (6.34%) |

*Aerial fitness (1), Pilates (3), Archery (1), Dance (1), Forest walking (1), Golf (2), Gym (1), Horse riding (2), Yoga (2), Other stretching (1), Walking (1), Off-road racing (1)

***Supplementary Table S6: Table showing the single most important barrier to PA***

|  | ***Total respondents***  ***(n=264)*** | ***Low PA***  ***(n=49)*** | ***Mod/High PA***  ***(n=176)*** |
| --- | --- | --- | --- |
| Fatigue | 125 (47.4%) | 21 (42.9%) | 84 (47.7%) |
| Pain | 42 (15.9%) | 12 (24.5%) | 24 (13.6%) |
| Lupus flare | 27 (10.3%) | 6 (12.2%) | 19 (10.8%) |
| Time | 22 (8.3%) | 2 (4.1%) | 17 (9.7%) |
| Motivation | 13 (4.9%) | 2 (4.1%) | 7 (4.0%) |
| Other illness | 11 (4.2%) | 3 (6.1%) | 4 (2.3%) |
| Other | 5 (1.9%) | 1 (2.0%) | 4 (2.3%) |
| Caregiving | 4 (1.5%) | 1 (2.04%) | 4 (1.8%) |
| Concern for lupus worsening | 4 (1.5%) | 0 | 4 (1.8%) |
| Concern for injury | 3 (1.1%) | 0 | 3 (1.3%) |
| Weather | 3 (1.1%) | 1 (2.0%) | 2 (1.1%) |
| Financial strains | 2 (0.8%) | 0 | 2 (1.1%) |
| Self-consciousness | 2 (0.8%) | 0 | 2 (1.1%) |
| Dislike of exercise | 1 (0.4%) | 0 | 1 (0.4%) |

***Supplementary Table S7:*** Table showing Median (IQR) age, BMI and diagnosis duration of respondents split by response (Yes or No) to barriers to PA.

| **Barrier** | **Response** | **N** | **Age (years)** | | **BMI (kg/m^2^)** | | **Diagnosis duration (years)** | |
| --- | --- | --- | --- | --- | --- | --- | --- | --- |
|  |  |  | **Median (IQR)** | **P-Value^a^** | **Median (IQR)** | **P-Value^a^** | **Median (IQR)** | **P-Value^a^** |
| Caregiving | No | 245 | 52 (40-59) | 0.4160 | 26.70 (23.40-32.20) | 0.1726 | 10 (4-20) | 0.4698 |
|  | Yes | 23 | 48 (35-58) |  | 28.60 (27.30-31.20) |  | 11 (6-23) |  |
| Concern for Injury | No | 227 | 52 (42-59) | 0.0131 | 27.00 (23.50-32.00) | 0.7979 | 10 (4-21) | 0.0063 |
|  | Yes | 41 | 46 (33-57) |  | 27.75 (23.15-31.75) |  | 7 (3-13) |  |
| Concern lupus worsens | No | 201 | 53 (42-59) | 0.0240 | 26.30 (23.30-31.10) | 0.0169 | 10 (5-23) | 0.0005* |
|  | Yes | 67 | 47 (35-58) |  | 28.85 (24.50-33.60) |  | 7 (2-16) |  |
| Dislike | No | 242 | 51 (41-59) | 0.5588 | 26.80 (23.40-31.20) | 0.0164 | 10 (4-20) | 0.9187 |
|  | Yes | 26 | 51 (33-59) |  | 31.65 (25.60-37.30) |  | 12 (3-24) |  |
| Fatigue | No | 32 | 55 (45-66) | 0.1023 | 24.35 (21.00-27.65) | 0.0014* | 10 (6-30) | 0.0894 |
|  | Yes | 236 | 51 (39-58) |  | 27.60 (23.60-32.40) |  | 10 (4-20) |  |
| Financial | No | 234 | 52 (40-59) | 0.3073 | 27.10 (23.60-31.60) | 0.9233 | 10 (4-20) | 0.5713 |
|  | Yes | 34 | 47 (39-58) |  | 27.55 (22.80-32.90) |  | 13 (4-22) |  |
| Flare/ symptoms of SLE | No | 113 | 54 (43-63) | 0.0007* | 26.00 (23.00-32.20) | 0.1542 | 13 (5-24) | 0.0012* |
|  | Yes | 155 | 48 (38-57) |  | 27.95 (23.60-31.70) |  | 8 (3-17) |  |
| Other illness | No | 178 | 50 (39-57) | 0.0544 | 27.05 (23.10-31.60) | 0.1578 | 10 (3-20) | 0.2429 |
|  | Yes | 90 | 53 (41-62) |  | 28.00 (24.10-32.90) |  | 10 (5-20) |  |
| Facilities | No | 248 | 51 (40-59) | 0.4596 | 27.30 (23.50-32.00) | 0.7602 | 10 (4-20) | 0.2949 |
|  | Yes | 20 | 47 (35-59) |  | 25.00 (23.55-35.35) |  | 8 (1-16) |  |
| Knowledge | No | 244 | 52 (41-59) | 0.2329 | 27.00 (23.40-31.70) | 0.3721 | 10 (4-20) | 0.4071 |
|  | Yes | 24 | 47 (35-57) |  | 28.20 (23.85-33.15) |  | 10 (3-17) |  |
| Motivation | No | 178 | 51 (43-59) | 0.2521 | 26.30 (23.10-31.10) | 0.0276 | 10 (4-22) | 0.7587 |
|  | Yes | 90 | 52 (35-58) |  | 27.80 (23.70-36.20) |  | 10 (3-19) |  |
| Time | No | 187 | 53 (42-61) | 0.0001* | 27.60 (23.60-32.90) | 0.1759 | 10 (4-20) | 0.4352 |
|  | Yes | 81 | 46 (36-53) |  | 26.30 (23.00-30.05) |  | 10 (4-21) |  |
| Pain | No | 102 | 52 (40-60) | 0.3075 | 25.60 (22.90-28.40) | 0.0003* | 10 (4-22) | 0.2324 |
|  | Yes | 166 | 51 (39-58) |  | 28.45 (23.80-33.35) |  | 10 (3-20) |  |
| Self-consciousness | No | 217 | 51 (41-60) | 0.0863 | 25.90 (23.20-30.40) | 0.0000* | 10 (4-20) | 0.3945 |
|  | Yes | 51 | 47 (35-58) |  | 31.10 (27.60-38.40) |  | 9 (3-20) |  |
| Weather | No | 208 | 52 (40-59) | 0.8671 | 27.30 (23.50-32.30) | 0.3976 | 10 (4-20) | 0.8036 |
|  | Yes | 60 | 51 (40-59) |  | 26.80 (22.90-30.10) |  | 10 (3-20) |  |
| ***^a^*** *P-Values are calculated from Mann-Whitney U test. Significant values at p<0.05 are in bold. If Bonferroni correction is applied due to multiple comparisons (P/15), significant p-value is p<0.00333, significant values are highlighted with asterisk.* | | | | | | | | |
